# Analyses of ovarian activity reveal repeated evolution of post-reproductive lifespans in toothed whales

**DOI:** 10.1038/s41598-018-31047-8

**Published:** 2018-08-27

**Authors:** Samuel Ellis, Daniel W. Franks, Stuart Nattrass, Thomas E. Currie, Michael A. Cant, Deborah Giles, Kenneth C. Balcomb, Darren P. Croft

**Affiliations:** 1 Centre for Research in Animal Behaviour, University of Exeter, Exeter, EX4 4QG UK; 2 Department of Biology, University of York, York, YO10 5DD UK; 3 Centre for Ecology and Conservation, University of Exeter, Penryn Campus, Penryn, Cornwall TR10 9FE UK; 4 Center for Whale Research, 355 Smugglers Cove Road, Friday Harbor, WA 98250 USA

## Abstract

In most species the reproductive system ages at the same rate as somatic tissue and individuals continue reproducing until death. However, females of three species – humans, killer whales and short-finned pilot whales – have been shown to display a markedly increased rate of reproductive senescence relative to somatic ageing. In these species, a significant proportion of females live beyond their reproductive lifespan: they have a post-reproductive lifespan. Research into this puzzling life-history strategy is hindered by the difficulties of quantifying the rate of reproductive senescence in wild populations. Here we present a method for measuring the relative rate of reproductive senescence in toothed whales using published physiological data. Of the sixteen species for which data are available (which does not include killer whales), we find that three have a significant post-reproductive lifespan: short-finned pilot whales, beluga whales and narwhals. Phylogenetic reconstruction suggests that female post-reproductive lifespans have evolved several times independently in toothed whales. Our study is the first evidence of a significant post-reproductive lifespan in beluga whales and narwhals which, when taken together with the evidence for post-reproductive lifespan in killer whales, doubles the number of non-human mammals known to exhibit post-reproductive lifespans in the wild.

## Introduction

Why a female should cease reproducing before their expected end of life is a long-standing question in evolutionary biology^1–3^. The taxonomic scarcity of this strategy suggests that it requires unusual selective pressures to evolve^4^. Comparative research has shown that females of only three species of mammal - humans (*Homo sapien*), killer whales (*Orcinus orca*) and short-finned pilot whales (*Globicephala macrorhynchus*) – are known to have a statistically significant post-reproductive lifespan in the wild^5^. Recent work has also suggested that females of a third toothed whale species, false killer whales (*Pseudorca crassidens*), may also have a post-reproductive lifespan^6^. Here we define post-reproductive lifespans as common and prolonged female survival after the cessation of reproduction, such that a female entering the adult population can expect to live a substantial period of her life post-reproductive^5,7^. We differentiate post-reproductive lifespans from non-adaptive brief and rare survival past last reproduction resulting from the usual processes of senescence^7^.

Given the logistical difficulties inherent in studying a predominantly oceanic taxon such as the toothed whales (*Odontoceti*) it is somewhat surprising that all but one of the species known to have post-reproductive lifespans are in this group. Recent research has suggested that the demographic consequences of certain social structures are important in life-history evolution^8^, and the evolution of post-reproductive lifespans in particular^4,9^. Toothed whales show a remarkable diversity of social and reproductive strategies^10,11^. The diversity of social structures and reproductive strategies in the toothed whales makes them an important target group to understand the evolution of post-reproductive lifespans.

Distinguishing post-reproductive lifespans from general declines in fecundity with age requires detailed data on reproduction and survival rates of the females in a population over their lifetime^12^. The difficulties of studying long-lived marine taxa mean that data meeting this requirement are rare for cetaceans. However, physiological data exist for many species: a consequence of mass mortality events and a tradition of using physiological data to infer life-history traits for parameterising conservation and management models^13^. In this study, we use these published physiological data to infer the rate and timing of reproductive senescence in female cetaceans. Using these data we analyse the rates of reproductive senescence as a population level trait and reveal the prevelance of post-reproductive life history in the toothed whales.

## Results

### Quantifying reproductive senescence

**Figure 1:**
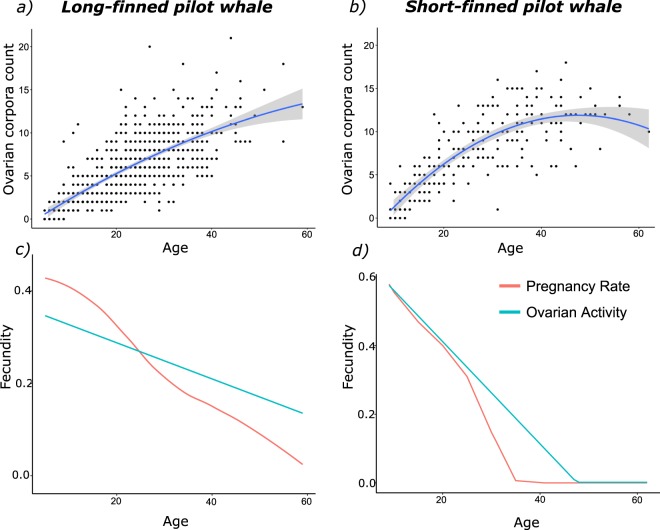
Calculation of age-specific fecundity for long-finned (**a**,**c**) and short-finned (**b**,**d**) pilot whales. Calculations for other species are shown in Supplementary 1. (**a**) and (**b**) Age-specific corpora counts in long-finned and short-finned pilot whales. Lines show fitted second order polynomial (with confidence intervals). Curves show that ovarian activity declines with age in both species, but that the decline is more pronounced in short-finned pilot whales than long-finned pilot whales. (**c**) and (**d**) Age-specific fecundity for long- and short- finned pilot whales calculated both from ovarian activity (the slope of the fitted polynomial (**a**,**b**)- blue line) and pregnancy rate (smoothed- red line). Both species show a decline in fecundity with age by both measures, however whereas short-finned pilot whale fecundity reaches 0 before the end of life, long-finned pilot whale fecundity does not. This is reflected in calculations of post-reproductive representation (a measure of post-reproductive lifespan, see text). Calculated from pregnancy rate short-finned pilot whales have a significant post-reproductive lifespan (PrR = 0.28^4^) whereas long-finned pilot whales do not (PrR = 0.02, calculated from^40,71^).

In cetaceans, female reproductive history can be inferred from anatomical examination of the ovaries^14^. After ovulation, the Graffian follicle, in which the ovum develops, degenerates first into a corpus luteum, and then into a corpus albicans^15,16^. In most Cetacea, these corpora remain present in the ovary and can therefore be used as an individual measure of past ovulation history. A decline in ovarian activity with age will result in slower formation of new corpora in older individuals (Fig. 1). The null expectation is that rate of corpora formation is constant throughout life, whereas a decrease in the rate of corpora formation with age indicates decreasing rates of ovulation as females get older: reproductive senescence.

We used published data of age-specific corpora counts to infer the timings and rate of reproductive senescence. These data are generated from anatomical examination of deceased whales (see methods). In a systematic search for age-specific corpora data in all 72 species of toothed whale we found sixteen species with age-specific corpora count data suitable for analysis: (see methods; Supplementary 1).

In ten of the sixteen species, a second degree polynomial best explained the relationship between corpora count and age showing that age-specific ovarian activity declined with age (e.g. Fig. 1; Table 1). The rate of reproductive senescence relative to the rate of somatic senescence in these ten species was inferred by comparing the standardised age-specific ovarian activity to standardised age (see methods; Table 1). Four species showed a rate of reproductive senescence greater than the rate of somatic senescence: beluga whale *Delphinapterus leucas* (relative rate of reproductive senescence = 1.69), narwhal *Monodon monoceros* (relative rate = 1.48), northern right-whale dolphin *Lissodelphis borealis* (relative rate = 1.14) and short-finned pilot whales (relative rate = 1.38). After the removal of an outlier the northern right-whale dolphin rate dropped to below one (relative rate = 0.75: this outlier is not removed for the calculation of population-level metrics, see below), but the other species results are qualitatively robust to the removal of outlying data.

**Table 1:** Reproductive senescence in toothed whales inferred from physiological analysis.

Common name	Species name	Age vs Corpora relationship	Relative rate of reproductive senescence	Phys-PrR stable population [shrinking population – growing population]	Conclusion
Beluga whale	*Delphinapterus leucas*	Polynomial	1.69	0.27* [0.19*–0.33*]	Reproductive senescence and post-reproductive lifespans
Narwhal	*Monodon monoceros*	Polynomial	1.48	0.24* [0.19*–0.29*]	Reproductive senescence and post-reproductive lifespans
Short-finned pilot whale	*Globicephala macrorhynchus*	Polynomial	1.38	0.15* [0.08*–0.22*]	Reproductive senescence and post-reproductive lifespans
Baird’s beaked whale	*Berardius bairdii*	Polynomial	0.87	0.01 [0.00–0.02]	Reproductive senescence
False killer whale	*Pseudorca crassidens*	Polynomial	0.77	0.03 [0.02–0.08]	Reproductive senescence
Long-finned pilot whale	*Globicephala melas*	Polynomial	0.62	0.01 [0.00–0.02]	Reproductive senescence
Northern right-whale dolphin	*Lissodelphis borealis*	Polynomial	1.14 (0.75)	0.03 [0.02–0.05]	Reproductive senescence
Pantropical spotted dolphin	*Stenella attenuata*	Polynomial	0.84	0.02 [0.01–0.03]	Reproductive senescence
Sperm whale	*Physeter macrocephalus*	Polynomial	0.64	0.00 [0.00–0.01]	Reproductive senescence
Spinner dolphin	*Stenella longirostris*	Polynomial	0.89	0.01 [0.01–0.02]	Reproductive senescence
Common bottlenose dolphin	*Tursiops truncatus*	1. Linear 2. Linear 3. Linear	n/a	n/a	No reproductive senescence
Melon-headed whale	*Peponocephala electra*	Linear	n/a	n/a	No reproductive senescence
Striped dolphin	*Stenella coeruleoalba*	Linear	n/a	n/a	No reproductive senescence
Atlantic white-sided dolphin	*Lagenorhynchus acutus*	No correlation	n/a	n/a	Corpora are not a good measure of ovarian activity.
Harbour porpoise	*Phocoena phocoena*	No correlation	n/a	n/a	Corpora are not a good measure of ovarian activity.
Short-beaked common dolphin	*Delphinus delphis*	No correlation	n/a	n/a	Corpora are not a good measure of ovarian activity.

The relative rate of reproductive senescence is calculated relative to somatic senescence using normalised data. A rate of exactly 1 would mean that ovarian activity is declining linearly with age. A rate of greater than 1 implies that ovarian activity is declining more slowly than somatic senescence. Rate in parentheses is the rate without a single outlying older individual. Phys-PrR (physiological post-reproductive representation) is the proportion of female years being lived by post-reproductive females in the population (those marked with an * are significantly different from 0).

In three species a linear relationship between corpora count and age suggests that there is no decrease in reproductive effort with age and therefore no reproductive senescence (Table 1). For three other species, we found no correlation between the number of corpora and age (adj-R^2^ ≤ 0.1; see Supplementary 1), suggesting that ovarian corpora are not a good measure of reproductive senescence in the species.

### Quantifying post-reproductive lifespans

Post-reproductive representation (PrR) is a population-level metric which calculates the proportion of adult female years in the population being lived by post-reproductive females. For example, in humans (without modern medical care) approximately 40% of adult female years are being lived by post-reproductive females (PrR = 0.443)^5^. PrR is typically performed on observational data, but here we calculate PrR in toothed whales from our physiological measures of rates of reproductive senescence (Phys-PrR). Assumptions around population growth rates (see methods) mean that the Phys-PrR is reported for a static population, a shrinking population and a growing population (as: static [shrinking - growing]).

**Figure 2:**
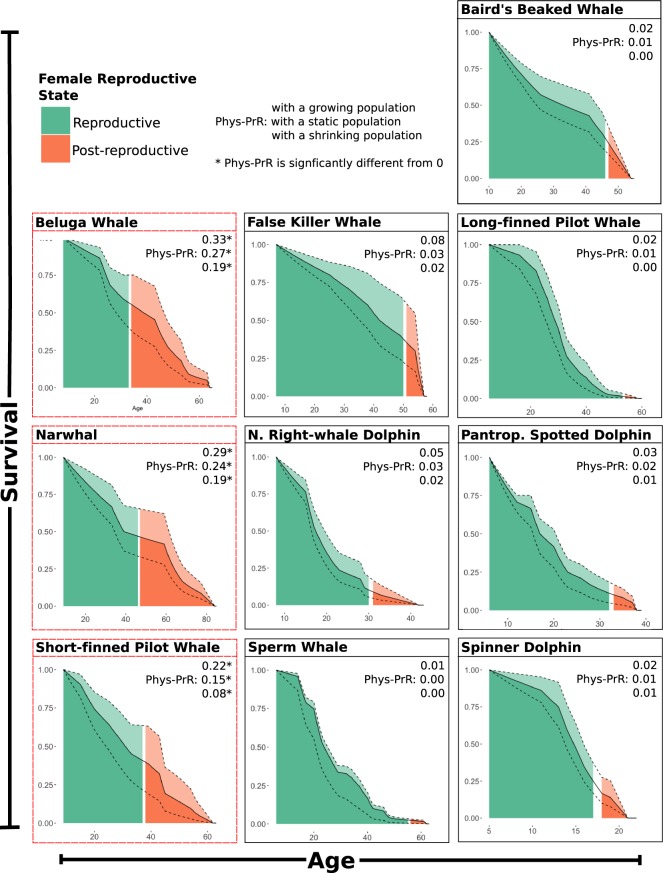
Female survival curves showing the predicted reproductive state of ten species of toothed whale. Females of three species: beluga whales, narwhals and short-finned pilot whale spend a significant proportion of their life post-reproductive. The age at which individuals become post-reproductive is defined based on the age at which 95% of population fecundity (measured as ovarian activity) has been completed. Green areas show when the females in the population are reproductively active, orange show when individuals are no longer reproductively active and therefore post-reproductive. The three curves represent different population change scenarios, the highest dashed curve represents a growing populating, the middle solid curve a static population and the lowest dashed curve a shrinking population (see methods for details). Physiological post-reproductive representation (Phys-PrR) is calculated based on age-specific ovarian activity, values denoted with an asterisk (*) are significantly different from 0, indicating that the species experiences post-reproductive lifespans.

Of the ten species that show a decline in reproductive activity with age (Table 1) in three a significant proportion of adult female years are being lived by post-reproductive females (Fig. 2): beluga whales (Phys-PrR = 0.27 [0.19–0.33], p < 0.001 [<0.001, <0.001]), narwhals (Phys-PrR = 0.24 [0.19–0.29], p < 0.001 [<0.001, <0.001]) and short-finned pilot whales (Phys-PrR = 0.15 [0.08–0.22], p < 0.001). For all other species, the proportion of post-reproductive adult females is not significantly different from 0 (Fig. 2). For three species, short-finned and long-finned pilot whales and false killer whales age-specific pregnancy data are also available, allowing us to validate our method (Fig. 1; Supplementary 2).

PrR calculated from observations of births or pregnancy data is comparable to Phys-PrR in both species of pilot whale and false killer whales (long-finned pilot whales, Phys-PrR = 0.02 [0.00, 0.02], PrR = 0.01, Fig. 1c; short-finned pilot whales, Phys-PrR = 0.15[0.08–0.22], PrR = 0.28), Fig. 1d; false killer whales – see Supplementary 2). This similarity demonstrates that our physiological measure reflects observed age-specific changes in reproductive activity. Moreover, using simulation approaches we demonstrate that our results are robust to potential errors in the estimation of whale ages (Supplementary 3).

**Figure 3:**
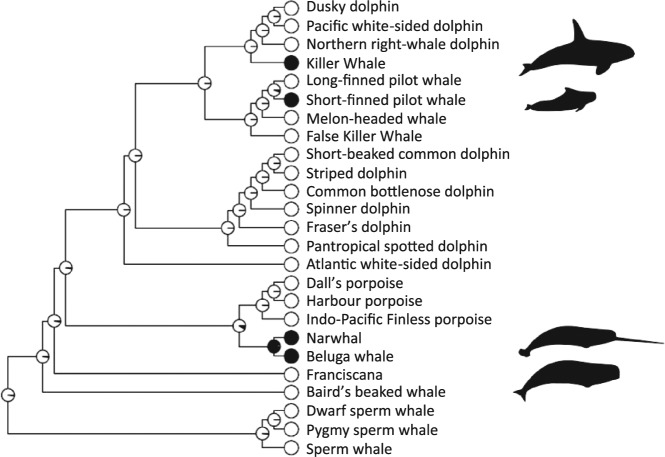
Phylogeny showing the evolution of post-reproductive lifespans in toothed whales (black; post-reproductive lifespans present, white; post-reproductive lifespans absent) for species in which data on the presence or absence of prolonged female post-reproductive lifespans are available. Pie charts at the nodes represent proportional probability that post-reproductive lifespans were present in ancestral species. Phylogenetic comparative methods (see methods) suggest that post-reproductive lifespans have evolved at least three times independently in Odontocete cetaceans. Species included are the 13 that show age-related changes in ovarian activity in this study and resident type killer whales which are well known to have a post-reproductive lifespan^17^ and 11 other species with records of reproduction in very old females (Table S2). Branch lengths are proportional to molecular change. Whale diagrams are adapted (cropped and the outline filled) from images by C Huh^72–75^ published under Creative Commons Licence 3.0- attribution share-alike unported (https://creativecommons.org/licenses/by-sa/3.0/).

We combined the physiological data on ovarian activity with other sources of information about toothed whale life-history to examine the evolution of post-reproductive lifespans in this clade. Phylogenetic ancestral state reconstruction reveals only one node, the common ancestor of the beluga and narwhal, with substantial support (proportional probability = 0.95) for the presence of post-reproductive lifespans in any ancestral species of any toothed whales. All the other nodes show very strong support (proportional probability > 0.9) for a lack of post-reproductive lifespans in ancestral species. Our results suggest that post-reproductive lifespans have evolved independently three times in the toothed whales: once at some point before the separation of the beluga and narwhal lineages, once in the lineage leading to short-finned pilot whales, and once in the lineage leading to killer whales (Fig. 3; Supplementary 4).

## Discussion

Using physiological data, we have found evidence of post-reproductive lifespans in three species of toothed whale: beluga whales, narwhals and short-finned pilot whales. To our knowledge this is the first record of population-level post-reproductive lifespans in beluga whales and narwhals, and supports previous suggestions of significant post-reproductive lifespans in short-finned pilot whales^5,17^. When taken together with the evidence for post-reproductive lifespans in killer whales from long-term individual based studies^5,18–20^ and non-invasive physiological studies^21^ our new findings effectively double the number of non-human mammals known to exhibit this unusual life-history strategy. Further, we found that post-reproductive lifespans are likely to result from at least three independent evolutionary transitions in the toothed whale lineage.

Recent work suggests that the evolution of post-reproductive lifespans in humans and killer whales is driven by changes in local relatedness across the lifespan^20,22^. In resident-ecotype killer whales (a salmon-eating killer whale population inhabiting north-east Pacific ocean), neither males or females disperse from their natal group: though males mate outside the group^23^. In ancestral humans dispersal is thought to have been female biased^24,25^. Under these demographic conditions selection will favour young females to invest resources in their own reproduction at the cost of reproduction in other group members^9,20^. Older females on the other hand can increase their inclusive fitness by aiding other group members to reproduce through grandmother and mother benefits^9,26^. Having a post-reproductive lifespan can be the optimal strategy when the inclusive fitness costs of reproductive conflict are combined with the inclusive fitness benefits of late-life helping^9,20^. Benefits to late-life helping and costs of reproductive conflict have been found in both humans and killer whales^20,22,26–28^. We predict that the two new species with a post-reproductive lifespan reported here will have demography that increases female within group relatedness as a function of age: either bisexual social philopatry with non-local mating or female dispersal with local mating^9^. Comparing the social structures of cetacean species with and without a significant female post-reproductive lifespan provides a unique opportunity to test the generality of demographic processes that are predicted to select for the evolution of post-reproductive lifespans^9,29^.

The limited information available on social structure in short-finned pilot whales, beluga and narwhal suggest that the population social structure may be based on bisexual philopatry. For example, genetic studies and observations at stranding events suggest that short-finned pilot whales live in mixed sex groups with males mating outside the group^30,31^. Similarly, both male and female beluga whales show high fidelity to natal summering feeding areas^32^, and both female and male (especially young males) are regularly found in association with close kin^33^. Narwhal societies appear to be focussed around matrilines while migrating to summer feeding grounds^34,35^. Thus, in all three species current evidence suggests patterns of demography, that may lead to an increase in females local relatedness to their group with age, much like in resident killer whales and ancestral humans^8,9^. Further work based on individual based longitudinal studies and or population genetic studies are needed to confirm these findings.

The social structures of toothed whales without a significant post-reproductive lifespan are varied (Supplementary 5). For example, female sperm whales live do not disperse and remain with their matrilineal unit throughout their life^10^. The males, in contrast, disperse at sexual maturity and are largely solitary, roving between female groups in search of mating opportunities^10^. This male-biased dispersal does not lead to the relatedness dynamics predicted to promote selection for female post-reproductive lifespans^9^. However, it is important to note that female relatedness to her group increasing with age does not presuppose the evolution of post-reproductive lifespans. For example, the available evidence suggests that long-finned pilot whales exhibit bisexual philopatry^36^ - much like short-finned pilot whales – and yet they do not have a significant post-reproductive lifespan. This highlights that it is not only demographic structures but also the balance of the costs of harming and the benefits of helping that may lead to the evolution of post-reproductive lifespans. Even within species there is considerable variation in social structure. Transient-ecotype killer whales (a mammal-eating population in the north-east Pacific), for example, form much smaller groups than resident-ecotype killer whales, and some males disperse from their natal group^37^. This would lead to very different relatedness structures in transient compared to resident-ecotype killer whale societies. It is unknown if transient-ecotype killer whales exhibit significant post-reproductive lifespans. Overall, there is a considerable amount still to be discovered about toothed whale social structure and life-history. Our results highlight the importance of the taxa for understanding the interplay between social behaviour and life history evolution.

In this study we use corpora count as a measure of fecundity. An advantage of using this physiological measure of fecundity is that the species in which ovarian activity ceases before the end of life are physiologically incapable of bearing in offspring (though this does not necessarily preclude them from lactating and nursing calves^30^). However, for many species females may stop reproduction prior to complete physiological reproductive senescence (measured via ovarian activity). For example, in rural Bangladesh women have their last child, on average, a decade before reproductive cesssation^38^. Detailed age-specific pregnancy data are rare for cetaceans, but do exist for short-finned pilot whales where, much like humans, the post-reproductive lifespan measured via pregnancy data is longer than the post-reproductive lifespan measured via ovarian activity (Fig. 1). This difference between a physiological measurement of reproduction and the direct observation of pregnancy may be a reason why our results differ from a recent analysis investigating the presence of post-reproductive lifespans in false killer whales^6^ (discussed in more detail: Supplementary 2). Our results are conservative in that the data captures age at last possible reproduction, rather than of last reproduction, and it is possible that detailed studies on age-specific pregnancy rates will reveal further cetacean species that exhibit a prolonged post-reproductive lifespan.

Studying the evolution of female post-reproductive life is hindered by its taxonomic rarity. Our physiological analyses gives new insight into life-history variation in cetaceans, and double the number of non-human mammals known to experience post-reproductive lifespans. This provides new opportunities to test the evolutionary origins and maintenance of post-reproductive lifespans in humans and toothed whales.

## Materials and Methods

### Data

We used published age-specific corpora counts to quantify the rate of reproductive senescence in female toothed whales. In Cetaceans, reproductive history can be inferred from anatomical examination of the ovaries^14^. After ovulation, the Graafian follicle, in which the ovum is develops, degenerates first into a corpus luteum, and then into a corpus albicans (hereafter collectively corpora) which persists in the ovary^39^. Corpora counts have been used to infer ovulation rate and other reproductive characteristics in a variety of Cetacean species (e.g.^30,39,40^). Here we use corpora counts as a measure of ovarian activity and not to estimate pregnancy rates which may differ from the corpora count^15,41,42^. In earlier studies corpora albicans and corpora atretica may not always have been properly distinguished^39^, however, as we are measuring ovarian activity, not ovulations *per se* this will not bias our results.

We undertook a thorough literature search for age-specific corpora count data on all 72 recognised species of Odontocetes^43^. Our criteria for data inclusion were: each female studied had a count of corpora and an independent estimate of age; female age structure was well represented; and that the data is presented in a clear format to be accurately obtained. Independent estimates of age were based on examination of dentine cemental layers in all species except narwhals, where the racemization of aspartic acid in the eye was used^44,45^. Recent research has shown that beluga whales deposit growth layer groups annually^46,47^. We therefore use growth layer group counts as our estimate of beluga whale age- though we note that as PrR is calculated as a proportion systematic age, estimation errors (doubled or halved for example) would not affect our conclusions. Appropriate data was found for sixteen species: Atlantic white-sided dolphin *Lagenorhynchus acutus*^48^, Baird’s beaked whale *Berardius bairdii*^49^, beluga whale *Delphinapterus leucas*^50^, common bottlenose dolphin *Tursiops truncatus*^51,52^, false killer whale *Pseudorca crassidens*^53^, harbour porpoise *Phocoena phocoena*^54^, long-finned pilot whale *Globicephala melas*^40^, melon-headed whale *Peponocephala electra*^55^, narwhal *Monodon monoceros*^45^, Northern right-whale dolphin *Lissodelphis borealis*^56^, Pantropical spotted dolphin *Stenella attenuata*^57^, short-beaked common dolphin *Delphinus delphis*^15^, short-finned pilot whale *Globicephala macrorhynchus*^30^, sperm whale *Physeter macrocephalus*^58^, spinner dolphin *Stenella longirostris*^59^ and striped dolphin *Stenella coeruleoalba*^57^. Previous work in resident killer whales (*Orcinus orca*) has documented significant post-reproductive lifespans using long term individual based observations^5,18–20^ and the post reproductive period has been confirmed using non-invasive hormonal samples^21^. Currently however, to our knowledge there are no published corpora count data on killer whales of a sufficiently large sample size for a robust test of the rate of reproductive senescence and a calculation of physiological PrR (but see^60^). Killer whales are not, therefore, included in our analysis of ovarian activity. Data were restricted to include only data from the age of first ovulation, i.e. the age with the first non-zero corpora count. All analysis was performed in R^61^ with the ggplot2 package used for producing the figures^62^.

It is important to note that throughout this study we refer to species, but our data is only based (with one exception) on a single population. For one species, the common bottlenose dolphin, data were available from three geographically distinct populations which we analyse independently. Data are also available from two false killer whale populations^53^, however we only use data from one population (Japan) as the second population (South Africa) may have been reproductively compromised^53^.

Our analysis is based on the assumption that corpora counts are a reliable measure of ovarian activity across the lifespan, which is supported by detailed examination of ovaries across a range of cetacean species^53,58,63^. For some species of cetacean however, there is evidence to suggest corpora may regress, and not persist indefinitely^15^ and in some cases there may be multiple eggs released at a single ovulation event^15^. However, there is no evidence of age-related changes in either poly-ovulation or regression of corpora, which could otherwise affect our analysis of age-dependent changes in ovarian activity. Indeed, for three species (short- and long-finned pilot whales, false killer whales) both pregnancy and corpora data are available and in both cases changes in pregnancy rate show a strikingly similar age-related pattern to changes in corpora deposition (Fig. 1c,d; Supplementary 2), validating our approach that ovarian activity (corpora count) can be used as a reliable measure of fecundity. To our knowledge this is the first population level examination of the relationship between corpora count and pregnancy rate.

### Quantifying reproductive senescence

A physiological decrease in fecundity with age in toothed whales will result in a lower rate of ovulation in older individuals. In populations with decreasing fecundity with age we therefore expect a second order relationship between ovarian activity and age, as older individuals are producing fewer new corpora per unit time. Reproductive senescence will be accompanied by a declining rate of ovarian activity with age. We fitted second order polynomials (which, inversed, decline in rate towards a peak) to each of the sixteen species to investigate this declining ovarian activity (e.g. Fig. 1). The change in ovarian activity with age is described by the slope of the fitted curve. A negative change in ovarian activity is an artefact of fitting a quadratic curve and was therefore treated as 0. We normalised both age and change in ovulation activity to between 0 and 1 to facilitate interspecies comparison.

We used AIC model comparison to investigate if the relationship between corpora count and age were best described by a 2^nd^ order polynomial or linear relationship. A linear relationship is our null assumption as it suggests that there is no decline in physiological reproductive activity through life.

We found a relationship between corpora count and age in thirteen of the sixteen species (detailed fit information; Supplementary 1). For three species we found no correlation between the number of corpora and age (adj-R2 ≤ 0.1), suggesting that either the data are too sparse or that ovarian corpora are not a good measure of reproductive senescence in the species. These three species are: Atlantic white-sided dolphin (adj-R^2^ = −0.02), harbour porpoise (adj-R^2^ = 0.07) and the short-beaked common dolphin (adj-R^2^ = 0.10). No further analysis was performed on these species.

### Calculating post-reproductive lifespans

For species with a decline in fecundity with age we then calculated their physiological post-reproductive representation (Phys-PrR). Post-reproductive representation is a population level measure describing the proportion of adult females years in the population that are being lived by post-reproductive females^12^. As our data are based on ovarian activity we measured the presence of physiologically post-reproductive females in the population (i.e. the proportion of females not ovulating).

The calculation of PrR is based on age-specific measures of survival and fecundity. We calculate age-specific survival from age-cohorts constructed from the original corpora data. Age-cohorts were constructed by making variable bin-widths starting at the oldest female in the study. We used these variable bin widths to construct monotonically decreasing age-cohorts, a pre-requisite for calculating survival from age-cohorts^64^. Bin widths were calculated in reverse: from the oldest individual. The oldest bin contains only the oldest female in the sample. The lower limit of the next bin was then selected to contain more than one whale, i.e. a greater number of females than the next oldest bin. This process continued until all females were assigned to a bin. In some cases, to fit the assumption of monotonically decreasing age cohorts the first age (youngest) bin for some species had to be smoothed to match the second youngest bin. This method will tend to underestimate late life survival, and therefore underestimate post-reproductive representation. Survival was then calculated from these age cohorts with survival assumed to be evenly spread through each age represented in a cohort. It should be noted that due to the low probability of sampling ‘rare’ ages of individuals, older whales are likely to be underrepresented in our data, further underestimating survival and the significance of the post-reproductive lifespan.

Calculating survival from age-cohort data assumes a stable population. If the population is not at equilibrium then calculation of survival, and therefore PrR, will be inaccurate. For example, in a growing population younger individuals will be overrepresented, underestimating late-life survival, and *vice versa*^64^. In the absence of detailed population growth parameters for most cetacean species, we model three population change scenarios in our calculation of Phys-PrR. Firstly, we assume a population at equilibrium, where population growth (*r*) = 0^64^. Secondly we assume a population in serious decline, *r* = −0.1, where the total population shrinks by 10% each year. We model the largest possible population growth scenario for each species, up to *r* = 0.1, given the age-structure of the data^64^. These values are comparable to the estimated population growth rates of cetacean populations. For example, at the peak of the modern sperm whale fishery between 1945 and 1975 the best estimate of global sperm whale population decline averaged approximately 2.67% (*r* = −0.027) per year (calculated from^13^). In contrast, North Atlantic humpback whales (*Megaptera novaeangliae*) may be recovering from very severe whaling at annual growth rate of 0.073–0.086^65^.

We used our measure of age-specific ovarian activity as a measure of fecundity. PrR is the summed life-expectancy in years after 95% of population fecundity has been completed (age M). Age M is independent of population change and therefore remains unchanged in the different growth scenarios. Because our data begin from maturity, age B (usually the age at which 5% of lifetime fecundity has been realised) is equal to the first age present in our data. We calculated Phys-PrR for each population change scenario for all ten species with evidence of reproductive senescence.

The significance of our PrR values was calculated by simulating the life-history of individuals based on the real survival and fecundity data. We calculate the estimated Phys-PrR of 1000 populations of 1000 individuals with reproductive senescence equal to somatic senescence^12^. The reported p values are the number of these simulated populations with a higher Phys-PrR than the real Phys-PrR. Significance is reported as the result of a two-tailed test.

It should be noted that these calculations are based on a stable and representative age structure. For some species (notably in this study sperm whales^13^ and beluga whales^66^) hunting pressures may have changed the demographics, with a bias to removing large (old) individuals from the population. For these species, this will lead to an underestimation of the frequency of post-reproductive females in the population, and therefore an underestimation of Phys-PrR.

### Phylogenetic ancestral state reconstruction

We combined the results of our Phys-PrR analysis with other published data on late-life reproduction to infer when post-reproductive lifespans have evolved in this clade using phylogenetic comparative methods. For this study we used a consensus tree created from the Bayesian posterior sample of 10,000 trees of the inferred phylogenetic relationships between cetacean species from the 10 k tree project^67^. This tree was pruned to leave only those species for which we have either physiological measures (n = 13) or other suitable records of reproduction in older females (n = 12; Fig. 3; Supplementary 4), resulting in a phylogenetic tree containing 25 species.

We used a continuous-time Markov chain method^68^ to model the evolution of post-reproductive lifespans as involving transitions between two states (post-reproductive lifespans present, and post-reproductive lifespans absent). This model has a single parameter, the instantaneous rate of change between these two states (transitions to and from post-reproductive lifespans are fixed to take the same value). We used the ancestral state estimation function in the R package “ape”^69^ in order to estimate the value for this rate parameter using maximum likelihood estimation. This approach allows us to infer the likely state of post-reproductive lifespans at ancestral nodes in the phylogeny given this model of evolution. These inferences are given as proportional probabilities (range: 0 to 1) and indicate whether ancestral species are likely to have had the trait under consideration.

### Ethics statement

All data used in this study are from published corpora counts from dissection of whale corpses. The corpses from each study come from a variety of sources (Supplementary 6). Some are from accidental deaths; five species data are from mass stranding events and four from by-catch in fisheries. Other data are from deliberate killing of whales; two species data are from aboriginal subsistence hunts, one from historical commercial whaling (sperm whales) and six from drive hunts in Japan and the Faroe Islands. The authors wish to state, in the strongest terms, that we in no way condone whaling as a data collection method. The data used here are from historical sources, collected by scientists working alongside commercial operations and no data were used from scientific whaling. We emphasise that terminal sampling is not the best way to collect data on reproductive senescence in cetaceans. Short, but especially long-term detailed demographic studies give much richer data for studying the relative rates of reproductive senescence, social structures and post-reproductive lifespans (e.g. killer whales in the Salish Sea^18,23,28,70^). In the absence of such published data for cetaceans we have made use of this historical physiological data, but highlight the need for, and value of, detailed individual based longitudinal demographic data in the future.

## Electronic supplementary material

Combined supplementary information

## Data Availability

All data used in this study are available in the publications referenced.
