# Supplementary material for: Analyses of ovarian activity reveal repeated evolution of post-reproductive lifespans in toothed whales
Source: Sci Rep. 2018 Aug 27;8:12833. doi: 10.1038/s41598-018-31047-8 (PMC6110730; doi:10.1038/s41598-018-31047-8)
Supplement: Supplementary file 1 — Combined supplementary information [file 41598_2018_31047_MOESM1_ESM.docx]

# Supplementary Materials:

**Analyses of ovarian activity reveal repeated evolution of post-reproductive lifespans in toothed whales**

**Authors:** Samuel Ellis^1^*, Daniel W. Franks^2^, Stuart Nattrass^2^, Thomas E. Currie^3^, Michael A. Cant^3^, Deborah Giles^4^, Kenneth C. Balcomb^4^, Darren P. Croft^1^.

**Author Affiliations**

^1.^ Centre for Research in Animal Behaviour, University of Exeter, Exeter, EX4 4QG, UK

^2.^ Department of Biology, University of York, York, YO10 5DD, UK

^3.^ Centre for Ecology and Conservation, University of Exeter, Penryn Campus, Penryn, Cornwall, TR10 9FE, UK

^4.^ Center for Whale Research, 355 Smugglers Cove Road, Friday Harbor, WA 98250, USA

* Correspondence and material requests to: s.ellis@exeter.ac.uk

**Keywords**

Post-reproductive lifespan, post-reproductive lifespans, life-history evolution, toothed whales

# Supplementary 1: Raw Data Plots and Model Fits

| **Species** | **Linear AIC** | **2nd Degree AIC** | **Conclusion** | **Source** |
| --- | --- | --- | --- | --- |
| **Atlantic white-sided dolphin**  *Lagenorhynchus acutus* | No correlation. | No correlation | Corpora are not a good measure of ovarian activity. | ^1^ |
| **Baird’s beaked whale**  *Berardius bairdii* | 148.6 | 147.0 | Reproductive senescence | ^2^ |
| **Beluga whale**  *Delphinapterus leucas* | 727.1 | 710.1 | Reproductive senescence and menopause | ^3^ |
| **Common bottlenose dolphin**  *Tursiops truncatus*  (3 populations) | 1. 583.5  2. 526.9  3. 175.2 | 1. 583.4  2. 523.0  3. 174.0 | No reproductive senescence | 1. ^4^  2. ^4^  3. ^5^ |
| **False killer whale**  *Pseudorca crassidens* | 293.4 | 292.1 | Reproductive senescence | ^6^ |
| **Harbour porpoise**  *Phocoena phocoena* | No correlation. | No correlation | Corpora are not a good measure of ovarian activity. | ^7^ |
| **Long-finned pilot whale**  *Globicephala melas* | 2792.5 | 2788.4 | Reproductive senescence | ^8^ |
| **Melon-headed whale**  *Peponocephala electra* | 236.5 | 236.6 | No reproductive senescence | ^9^ |
| **Narwhal**  *Monodon monoceros* | 258.8 | 253.4 | Reproductive senescence and menopause | ^10^ |
| **Northern right-whale dolphin**  *Lissodelphis borealis* | 355.2 | 345.2 | Reproductive senescence | ^11^ |
| **Pantropical spotted dolphin**  *Stenella attenuata* | 624.8 | 623.2 | Reproductive senescence | ^12^ |
| **Short-finned pilot whale**  *Globicephala macrorhynchus* | 935.5 | 899.9 | Reproductive senescence and menopause | ^13^ |
| **Short-beaked common dolphin**  *Delphinus delphis* | No correlation. | No correlation | Corpora are not a good measure of ovarian activity. | ^14^ |
| **Sperm Whale**  *Physeter macrocephalus* | 3807.1 | 3788.3 | Reproductive senescence | ^15^ |
| **Spinner Dolphin**  Stenella longirostris | 820.1 | 816.4 | Reproductive senescence | ^16^ |
| **Striped Dolphin**  *Stenella coeruleoalba* | 632.0 | 633.0 | No reproductive senescence | ^12^ |

Table S1. AIC fits of linear and 2^nd^ order polynomial linear models on the raw corpora data.


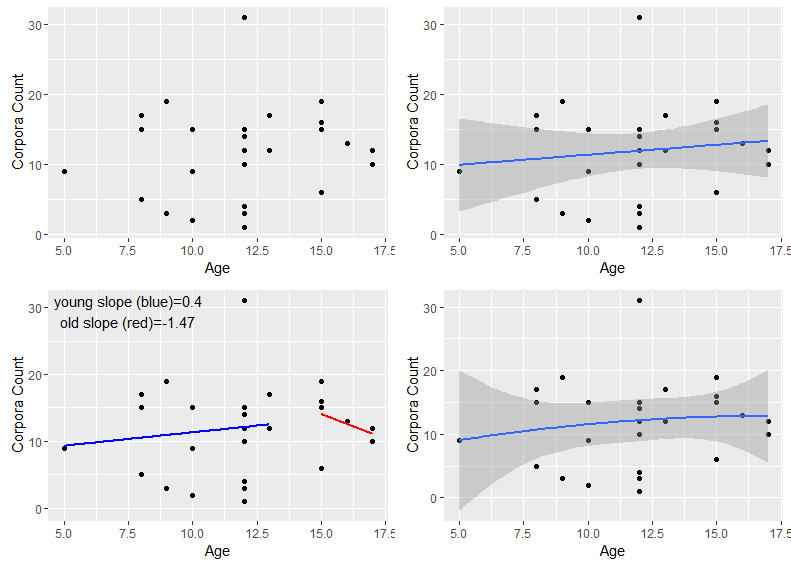


**Fig. S1.1.** Atlantic white-sided dolphin raw data and fits. Top left figure shows the raw data, top right a linear fit, bottom left two linear fits (blue= first two thirds of life, red = last third of life) and bottom right the fit of a 2^nd^ order polynomial. See table S1 for AIC model fits and data origin.


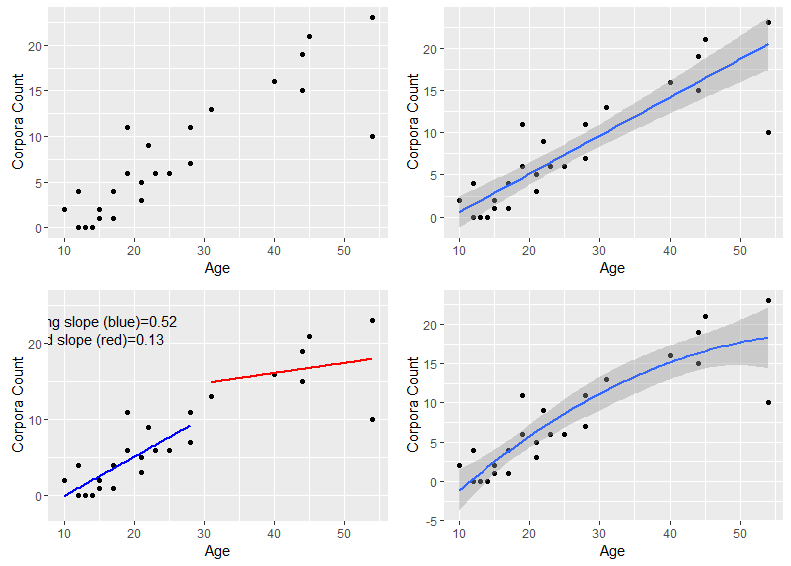


**Fig. S1.2.** Baird’s beaked whale raw data and fits. Top left figure shows the raw data, top right a linear fit, bottom left two linear fits (blue= first two thirds of life, red = last third of life) and bottom right the fit of a 2^nd^ order polynomial. See table S1 for AIC model fits and data origin.


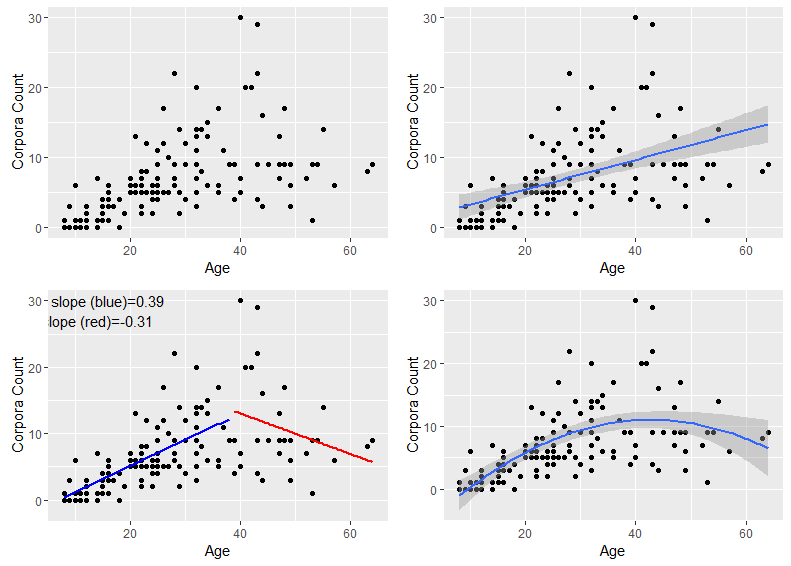


**Fig. S1.3.** Beluga whale raw data and fits. Top left figure shows the raw data, top right a linear fit, bottom left two linear fits (blue= first two thirds of life, red = last third of life) and bottom right the fit of a 2^nd^ order polynomial. See table S1 for AIC model fits and data origin.


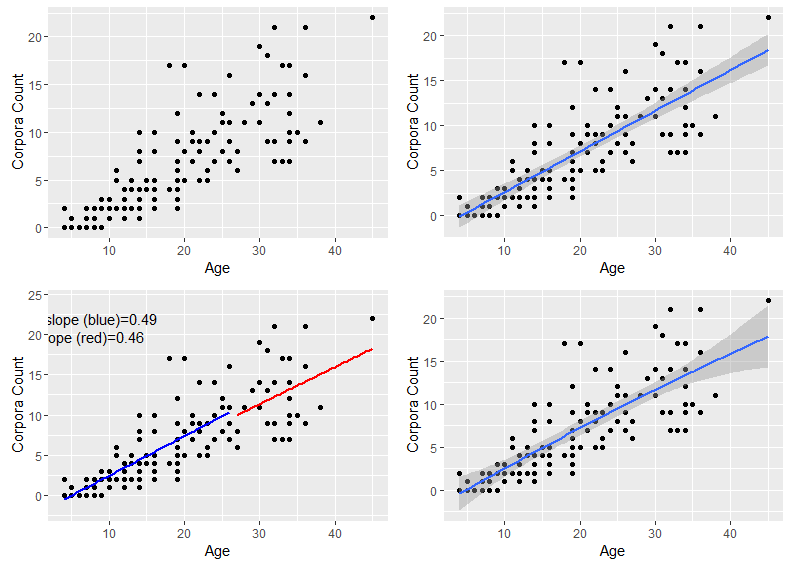


**Fig. S1.4** Common bottlenose dolphins from the Korean strait raw data and fits. Top left figure shows the raw data, top right a linear fit, bottom left two linear fits (blue= first two thirds of life, red = last third of life) and bottom right the fit of a 2^nd^ order polynomial. See table S1 for AIC model fits and data origin.


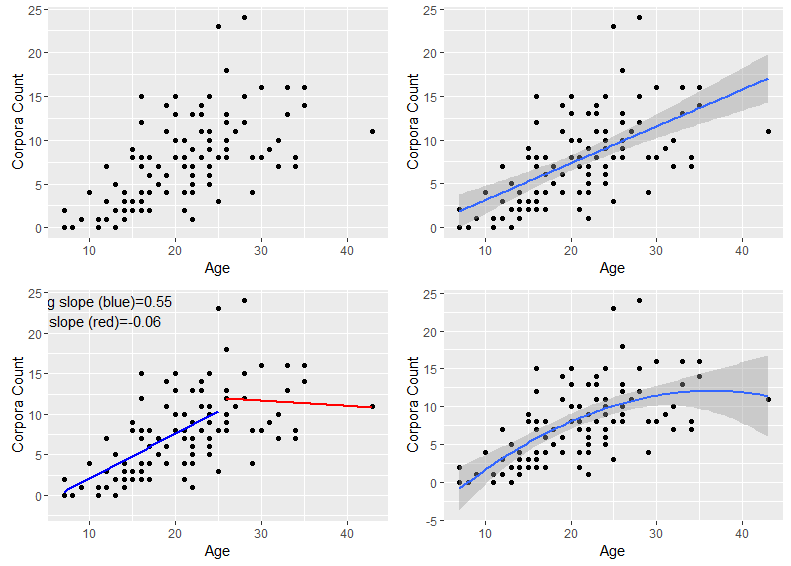


**Fig. S1.5.** Common bottlenose dolphins from the sea of Japan raw data and fits. Top left figure shows the raw data, top right a linear fit, bottom left two linear fits (blue= first two thirds of life, red = last third of life) and bottom right the fit of a 2^nd^ order polynomial. See table S1 for AIC model fits and data origin.


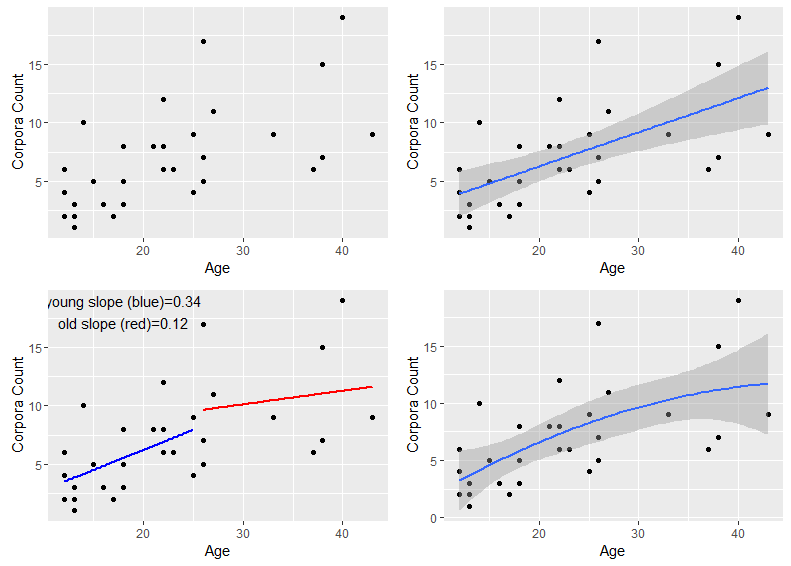


**Fig. S1.6.** Common bottlenose dolphins from South Africa raw data and fits. Top left figure shows the raw data, top right a linear fit, bottom left two linear fits (blue= first two thirds of life, red = last third of life) and bottom right the fit of a 2^nd^ order polynomial. See table S1 for AIC model fits and data origin.


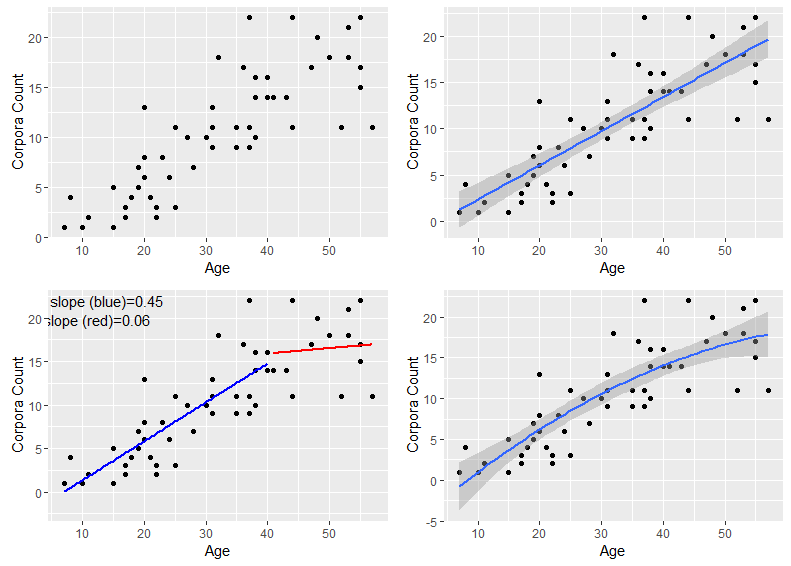


**Fig. S1.7.** False killer whale raw data and fits. Top left figure shows the raw data, top right a linear fit, bottom left two linear fits (blue= first two thirds of life, red = last third of life) and bottom right the fit of a 2^nd^ order polynomial. See table S1 for AIC model fits and data origin.


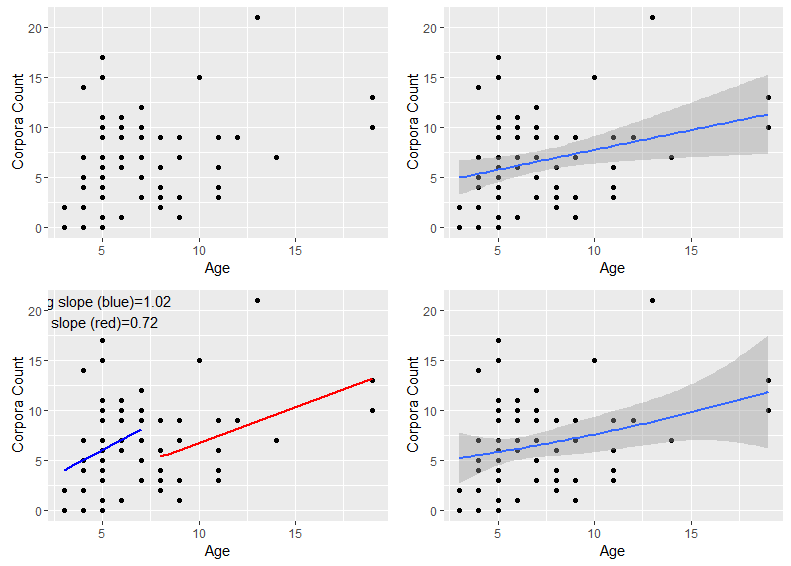


**Fig. S1.8.** Harbour Porpoise raw data and fits. Top left figure shows the raw data, top right a linear fit, bottom left two linear fits (blue= first two thirds of life, red = last third of life) and bottom right the fit of a 2^nd^ order polynomial. See table S1 for AIC model fits and data origin.


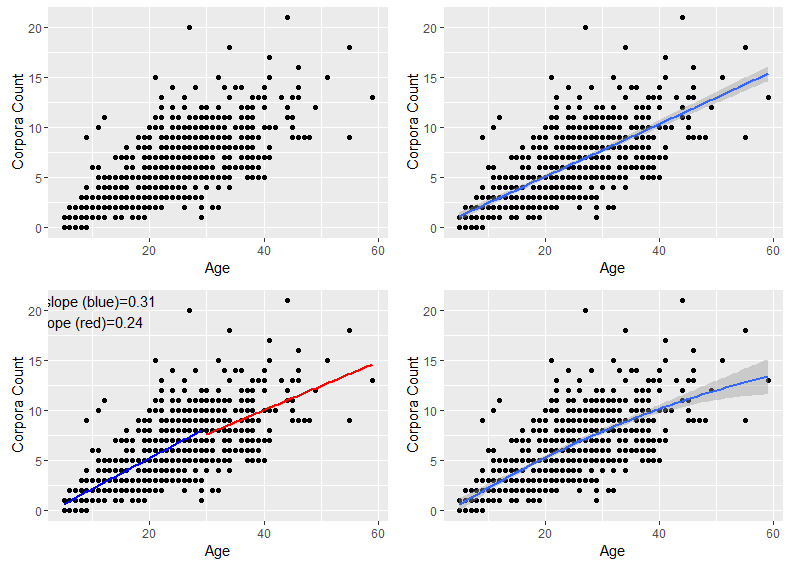


**Fig. S1.9.** Long-finned pilot whale raw data and fits. Top left figure shows the raw data, top right a linear fit, bottom left two linear fits (blue= first two thirds of life, red = last third of life) and bottom right the fit of a 2^nd^ order polynomial. See table S1 for AIC model fits and data origin.


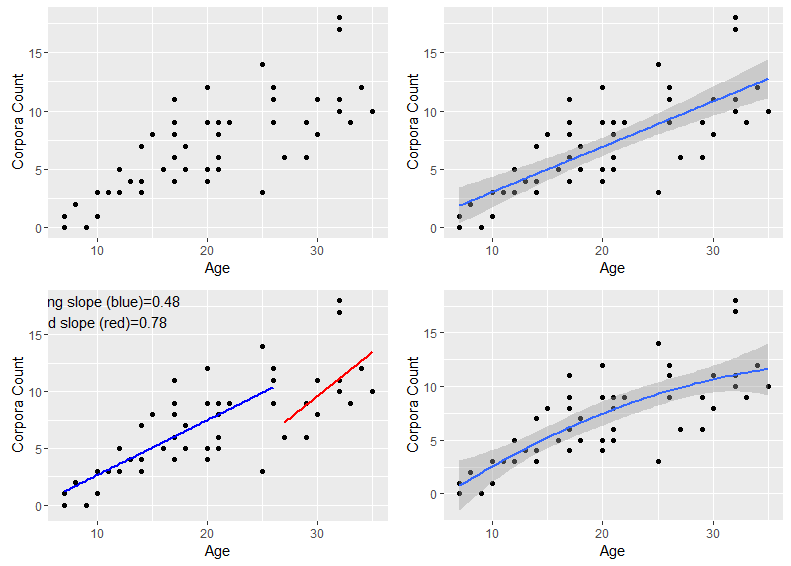


**Fig. S1.10.** Melon-headed whale whale raw data and fits. Top left figure shows the raw data, top right a linear fit, bottom left two linear fits (blue= first two thirds of life, red = last third of life) and bottom right the fit of a 2^nd^ order polynomial. See table S1 for AIC model fits and data origin.


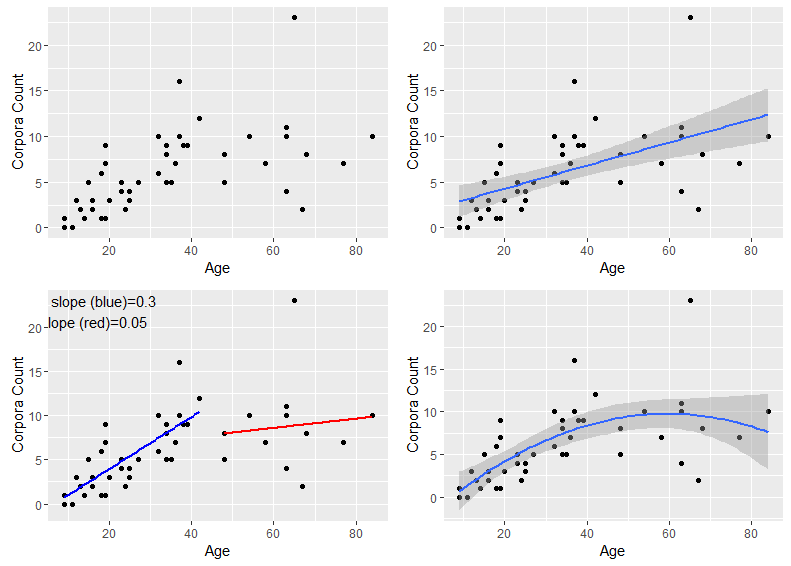


**Fig. S1.11.** Narwhal raw data and fits. Top left figure shows the raw data, top right a linear fit, bottom left two linear fits (blue= first two thirds of life, red = last third of life) and bottom right the fit of a 2^nd^ order polynomial. See table S1 for AIC model fits and data origin.


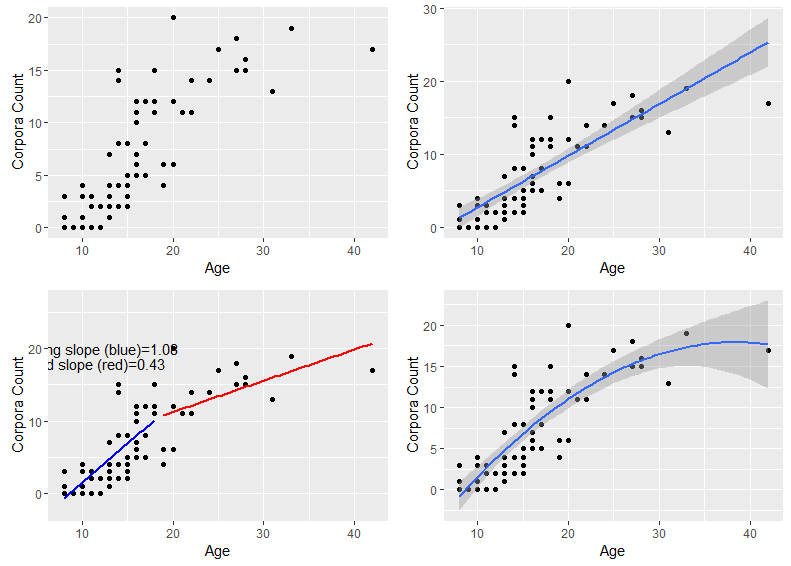


**Fig. S1.12.** Northern right-whale dolphin raw data and fits. Top left figure shows the raw data, top right a linear fit, bottom left two linear fits (blue= first two thirds of life, red = last third of life) and bottom right the fit of a 2^nd^ order polynomial. See table S1 for AIC model fits and data origin.


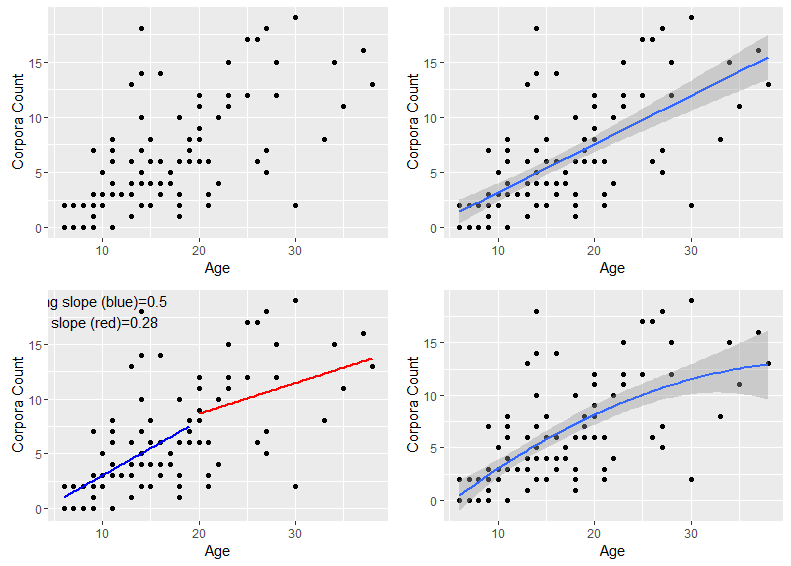


**Fig. S1.13.** Pantropical spotted dolphin raw data and fits. Top left figure shows the raw data, top right a linear fit, bottom left two linear fits (blue= first two thirds of life, red = last third of life) and bottom right the fit of a 2^nd^ order polynomial. See table S1 for AIC model fits and data origin.


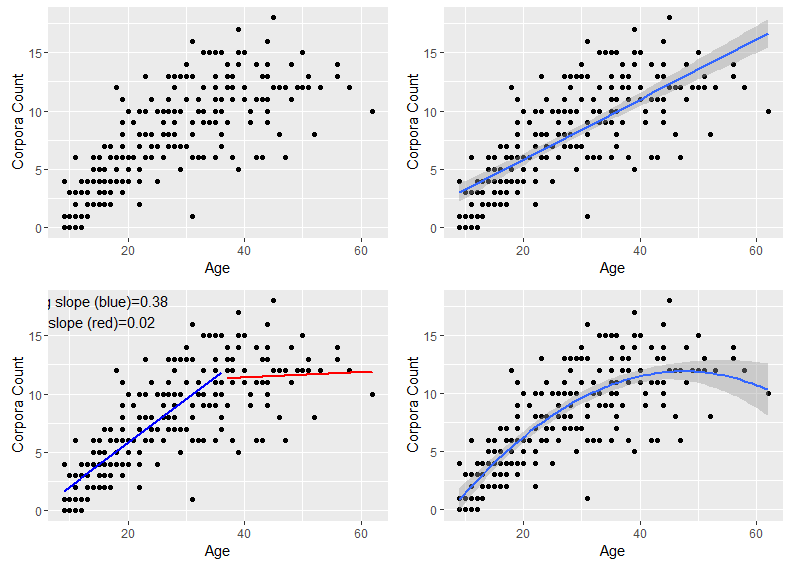


**Fig. S1.14.** Short-finned pilot whale raw data and fits. Top left figure shows the raw data, top right a linear fit, bottom left two linear fits (blue= first two thirds of life, red = last third of life) and bottom right the fit of a 2^nd^ order polynomial. See table S1 for AIC model fits and data origin.


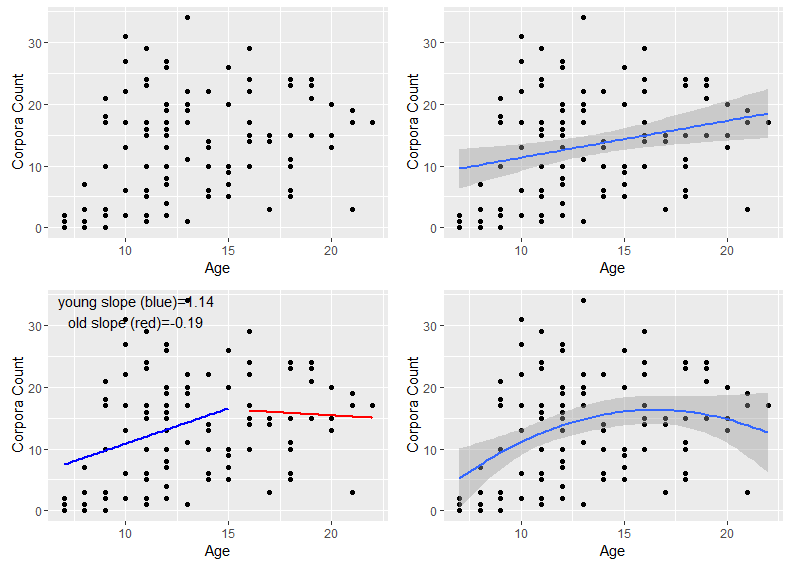


**Fig. S1.15.** Short-beaked common dolphin raw data and fits. Top left figure shows the raw data, top right a linear fit, bottom left two linear fits (blue= first two thirds of life, red = last third of life) and bottom right the fit of a 2^nd^ order polynomial. See table S1 for AIC model fits and data origin.


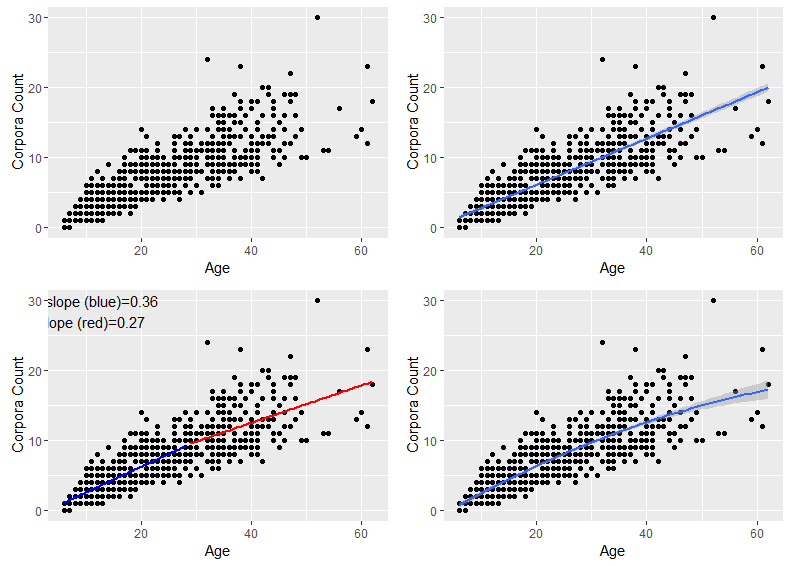


**Fig. S1.16.** Sperm whale raw data and fits. Top left figure shows the raw data, top right a linear fit, bottom left two linear fits (blue= first two thirds of life, red = last third of life) and bottom right the fit of a 2^nd^ order polynomial. See table S1 for AIC model fits and data origin.


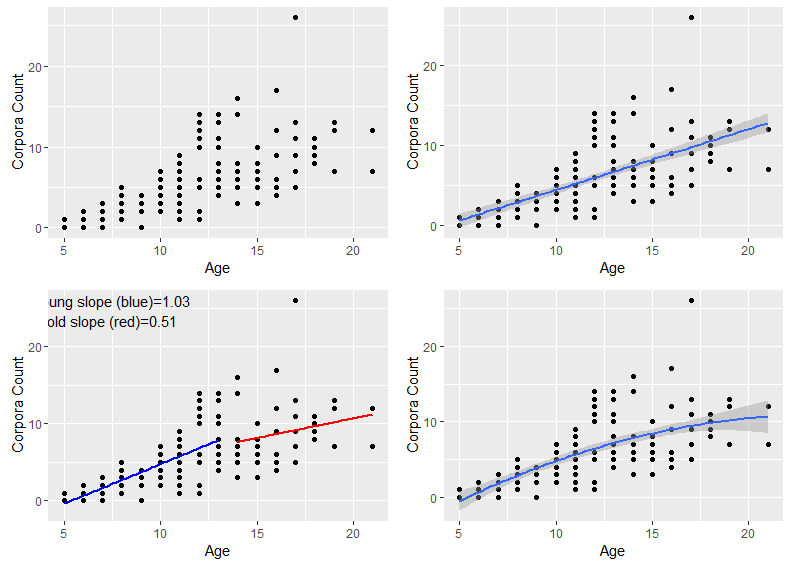


**Fig. S1.17.** Spinner Dolphin raw data and fits. Top left figure shows the raw data, top right a linear fit, bottom left two linear fits (blue= first two thirds of life, red = last third of life) and bottom right the fit of a 2^nd^ order polynomial. See table S1 for AIC model fits and data origin.


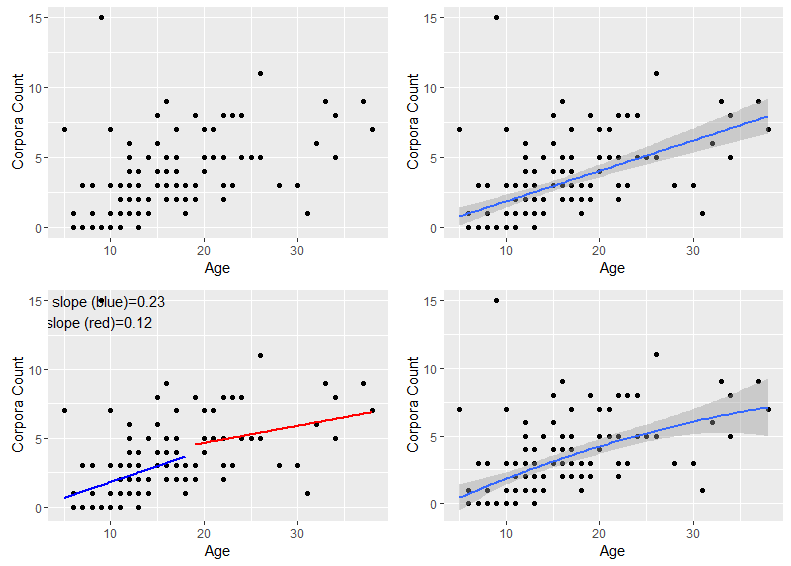


**Fig. S1.18.** Striped dolphin raw data and fits. Top left figure shows the raw data, top right a linear fit, bottom left two linear fits (blue= first two thirds of life, red = last third of life) and bottom right the fit of a 2^nd^ order polynomial. See table S1 for AIC model fits and data origin.

**References**

1. Sergeant, D. E., St. Aubin, D. J. & Geraci, J. R. Life history and northwest Atlantic status of Atlantic white-sided dolphin *Lagenorhynchus actus*. *Cetology* **30,** 1–12 (1980).
2. Kasuya, T., Brownell Jr, R. L. & Balcomb, K. C. Life history of Baird’s beaked whales. *Rep. Int. Whal. Comm.* **47,** 969–979 (1997).
3. Suydam, R. S. *Age, growth, reproduction, and movements of beluga whales (Delphinapterus leucas) from the eastern Chukchi Sea*. *PhD Thesis* (2009).
4. Kasuya, T., Izumisawa, Y., Komyo, Y., Ishino, Y. & Maejima, Y. Life history parameters Bottlenose dolphins off Japan. *IBI Reports* **7,** 71–107 (1997).
5. Cockcroft, V. G. & Ross, G. J. B. Age, growth, and reproduction of Bottlenose dolphins *Tursiops truncatus* from the east coast of southern Africa. *Fish. Bull.* **88,** 289–302 (1990).
6. Ferreira, I. M., Kasuya, T., Marsh, H. & Best, P. B. False killer whales (*Pseudorca crassidens*) from Japan and South Africa: Differences in growth and reproduction. *Mar. Mammal Sci.* **30,** 64–84 (2014).
7. Murphy, S. *et al.* Reproductive failure in UK harbour porpoises *Phocoena phocoena*: Legacy of pollutant exposure? *PLoS One* **10,** 1–32 (2015).
8. Amano, M. *et al.* Life history and group composition of melon-headed whales based on mass strandings in Japan. *Mar. Mammal Sci.* **30,** 480–493 (2014).
9. Martin, A. R. & Rothery, P. Reproductive parameters of female long-finned pilot whales (*Globicephala melas*) around the Faroe Islands. *Rep. Int. Whal. Comm.* 263–304 (1993).
10. Garde, E. *et al.* Life history parameters of narwhals (*Monodon monoceros*) from Greenland. *J. Mammal.* **96,** 866–879 (2015).
11. Ferrero, R. C. & Walker, W. A. Growth and reproduction of the northern right whale dolphin  *Lissodelphis borealis*  in the offshore waters of the North Pacific Ocean. *Can. J. Zool.* **71,** 2335–2344 (1993).
12. Kasuya, T. Reconsideration of life history parameters of the spotted and striped dolphin based on cemental layers. *Sci. Reports Whales Res. Institute, Tokyo* **28,** (1976).
13. Kasuya, T. & Marsh, H. Life history and reproductive biology of the short-finned pilot whale, *Globicephala macrorhynchus*, off the Pacific coast of Japan. *Rep. Int. Whal. Comm.* 259–310 (1984).
14. Dabin, W., Cossais, F., Pierce, G. J. & Ridoux, V. Do ovarian scars persist with age in all Cetaceans: New insight from the short-beaked common dolphin (Delphinus delphis Linnaeus, 1758). *Mar. Biol.* **156,** 127–139 (2008).
15. Ohsumi, S. Reproduction of the sperm whale in the north-west Pacific. *Sci. Reports Whales Res. Institute, Tokyo* **19,** 1–35 (1965).
16. Perrin, W. F., Holts, D. B. & Miller, R. B. Growth and reproduction of the eastern spinner dolphin, a geographical form of *Stenella longirostris* in the Eastern Tropical Pacific. *Fish. Bull.* **75,** 725–750 (1977).

# Supplementary 2: False Killer Whales

A comparison of reproductive senescence in the false killer whale calculated from corpora data (this paper, data from [1]) and calculated from pregnancy data (data from [2]). Both papers refer to the same samples from the same populations, we use only the data from Japan for reasons outlined in the methods. Pregnancy is calculated as the proportion of females of a given age (or in this case, age category) who are pregnant. As it is reported as a proportion it is vulnerable to small samples strongly influencing the reported pregnancy rate. To account for this, fecundity data were smoothed by weighting the magnitude of fecundity [3] change between age *x* and age *x*+1 by the number of individuals sampled at *x*+1. By using this smoothing method, and in contrast to what has been reported previously [2], we found no evidence of a significant post-reproductive lifespan in false killer whales using either pregnancy (PrR= 0.058), or ovarian activity data (Phys-PrR= 0.03 [0.02-0.08]). In both cases our measures are conservative and by controlling for small sample sizes in the false killer whales we extend the period of fecundity. In the raw data no pregnancy are reported after the age of 44.25, however the drop of fecundity from 25% at age 38.25- 44.25 of females pregnant to 0% after this age is very steep (especially given that there are only 3 females observed between the ages of 44.25 and 50.25). The measures we report are unlikely to return false positives, more data are needed to establish the extent and frequency of post-reproductive life in false killer whales.

**
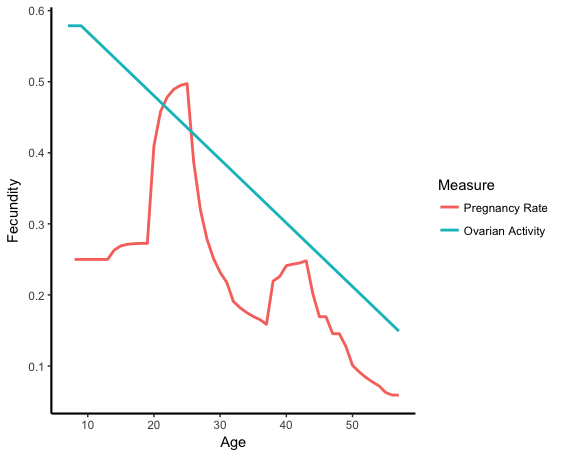
**

Figure S2. Relationship between age and two measures of fecundity in False Killer Whales.

**References**

1. Ferreira, I. M., Kasuya, T., Marsh, H. & Best, P. B. 2014 False killer whales (*Pseudorca crassidens*) from Japan and South Africa: Differences in growth and reproduction. *Mar. Mammal Sci.* **30**, 64–84. (doi:10.1111/mms.12021)

2. Photopoulou, T., Ferreira, I. M., Kasuya, T., Best, P. B. & Marsh, H. 2017 Evidence for a postreproductive phase in female false killer whales Pseudorca crassidens. *Front. Zool.* **14**, 1–24. (doi:10.1186/s12983-017-0208-y)

3. Ellis, S. et al. 2018 Postreproductive lifespans are rare in mammals. *Ecol. Evol.* , 1–13. (doi:10.1002/ece3.3856)

# Supplementary 3: Modelling the effect of errors in age estimation

The age of whales in this study were estimated from anatomical features (teeth ^1,2,3,4,5,6,7,8,9,10,11,12,13,9^ or lens amino acids^14^). Error is inherent in such estimates. We performed a series of simulations to understand the effect that errors in age estimation would have on calculations of Phys-PrR. Systematic error (for example doubled or halved ages) would have no effect on the calculation of Phys-PrR which is based on proportion of observed lifespan- not actual lifespan. Random errors in age estimation however have the potential to effect the estimate of PrR in unpredictable ways.

To understand these sources of error we simulated error in our estimation of ages. We took the raw data (supplementary 1) for each species. For each individual (and therefore each corpora count) we gave their individual some form of error (described below). We then continued the calculation of Phy-PrR s before for the permuted population. We repeated this process 1000 times and examined the distribution of age error Phys-PrRs compared to the observer Phys-PrR value. Similar values of ‘error’ Phys-PrR and ‘observed’ PrR suggest that the observed value of Phys-PrR is robust to errors in estimation of whale ages.

We present the results of three kinds of error:

1. *Error within a 5 year window*. For each age, a randomised age was chosen from a uniform 5 year distribution around the recorded age (i.e. 2. 5 years in + and – from the original point). For example, for a whale with an observed age of 10 the window will extend from 7.5 to 12.5. The lower window was limited at 0.
2. *Error within 10% of species maximum lifespan*. We took the 10% of the age of the maximum of age as the window width (window width). For each age, a randomised age is chosen from a uniform distribution of the window width (i.e. +/- 0.5 x the window width). For example, for a species with a maximum age of 100 the window will be 5 years in either direction of each observed age. This is randomisation scales with the lifespan of the species. As above, the lower window was limited at 0.
3. *Error within 10% of the observed age*. For each age, we choose a randomised age from a uniform distribution from a window of 10% of the current age. For example, if the observed age was 50, the window will be 5 years in each direction, from 45 to 55. Under this error structure, absolute error will be greater at older ages. As in the above error forsm, the lower window is limited at 0.

Results are presented below (Error 1 five year window fig S 5.1, Error 2 10% max lifespan fig S 5.2, 10% Error 3 10% observed age S 5.3). In general error estimation makes little qualitative difference to the estimate of Phys-PrR. Importantly, under all error structures beluga whale, narwhal and short-finned pilot whales have PrRs consistently well above 0, and higher than the other species tested.

**Figure S3.1**

Phys-PrR calculated from 1000 populations with error in age estimation introduced with a five year window of the determined age. For all figures, red line shows the observed Phy-PrR. Solid blue line is the mean of the error estimated Phys-PrRs, dashed blue lines show +/- two standard deviations of that mean. The PrRs of beluga whales, narwhals and short-finned pilot whales are consistently high with this form of age error.


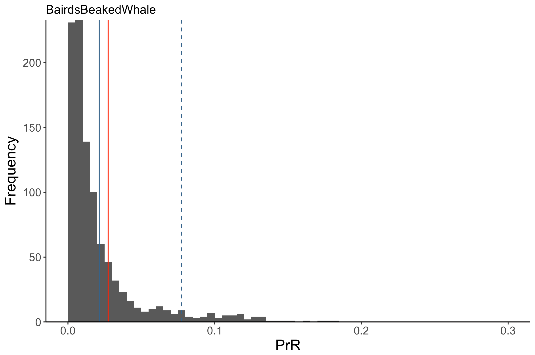

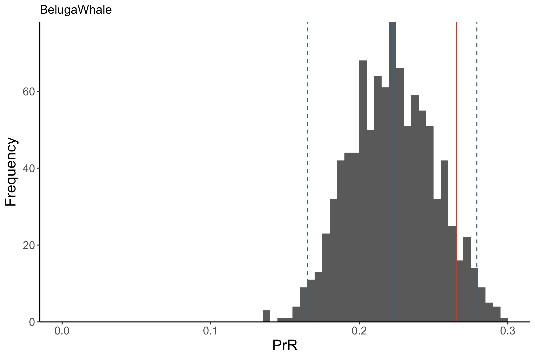

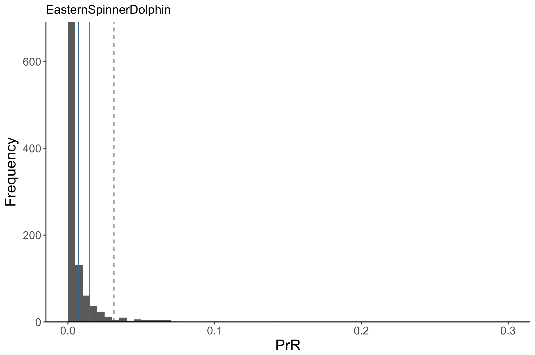

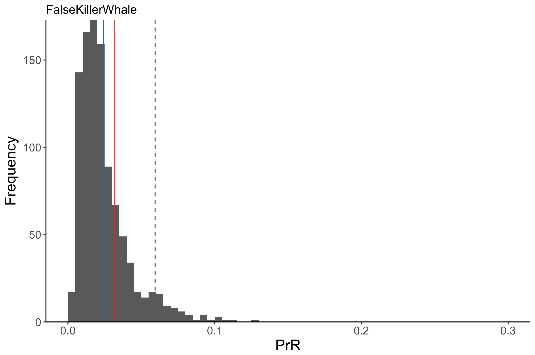

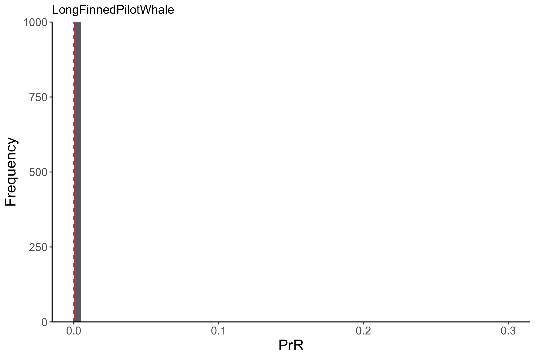

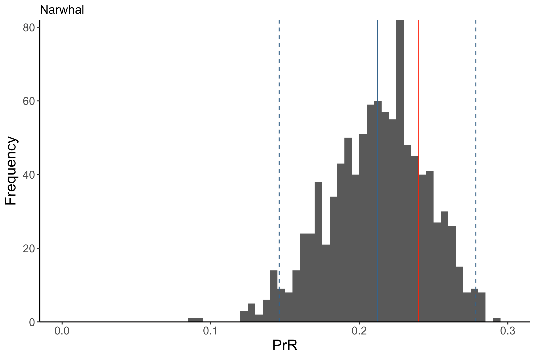

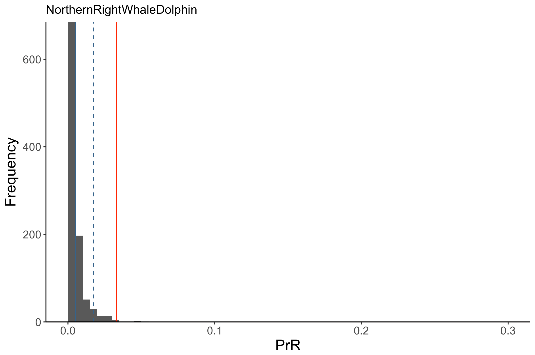

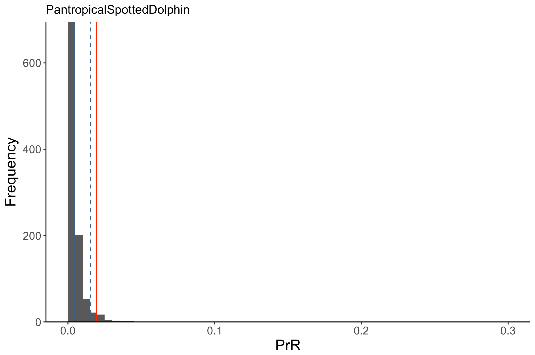

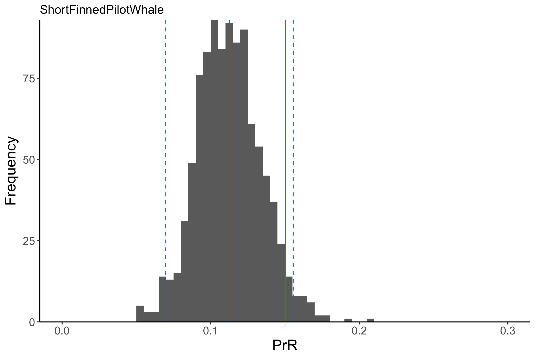

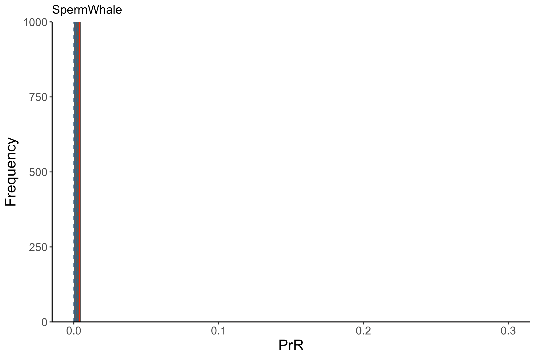


**Figure S3.2**

Phys-PrR calculated from 1000 populations with error in age estimation introduced with a ten percent window of the maximum age found in the species. For all figures, red line shows the observed Phy-PrR. Solid blue line is the mean of the error estimated Phys-PrRs, dashed blue lines show +/- two standard deviations of that mean. The PrRs of beluga whales, narwhals and short-finned pilot whales are consistently high with this form of age error.


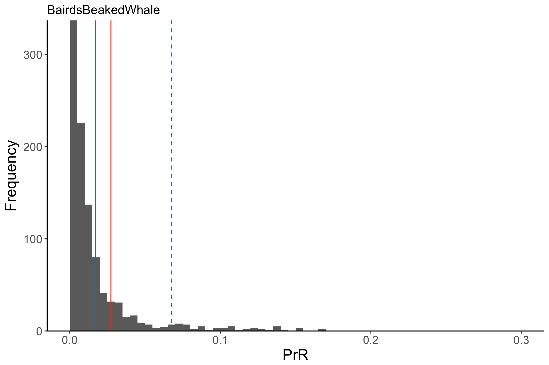

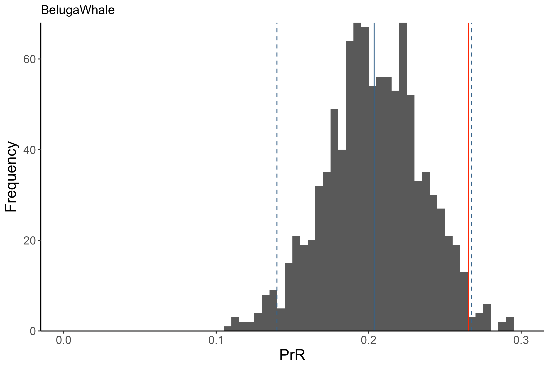

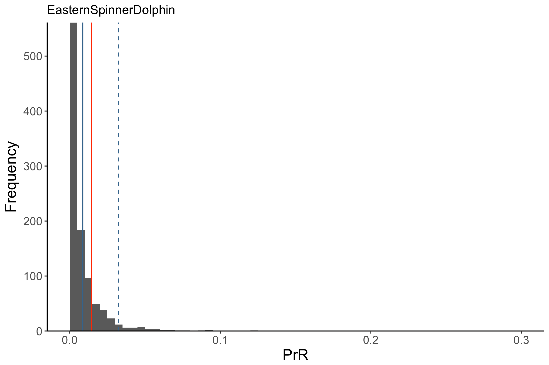

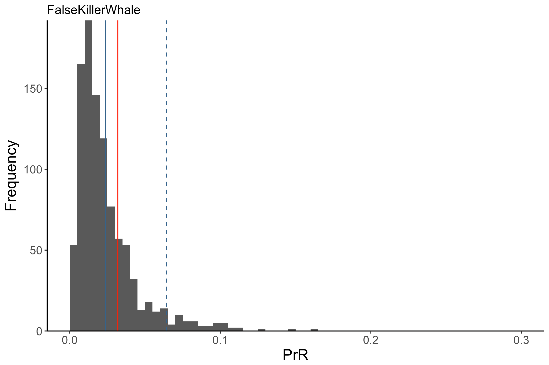

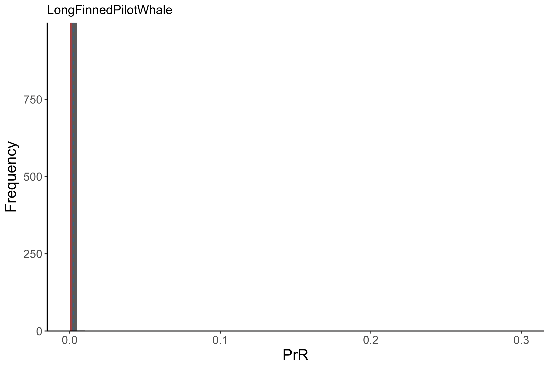

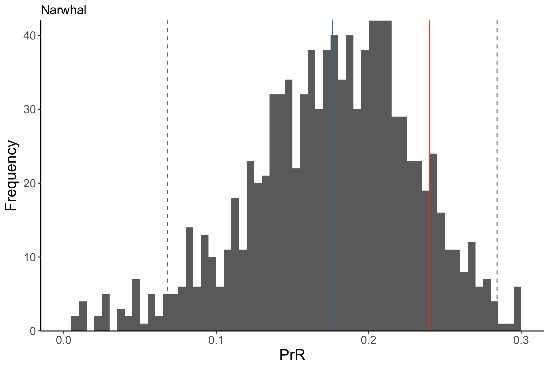

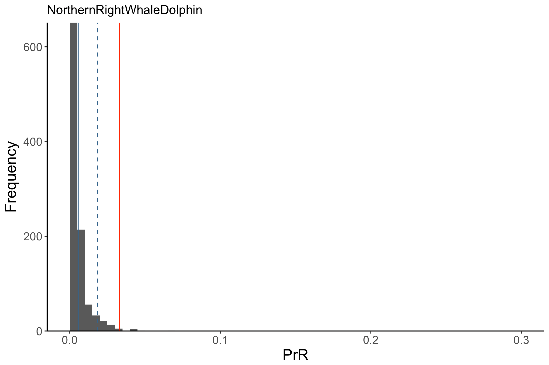

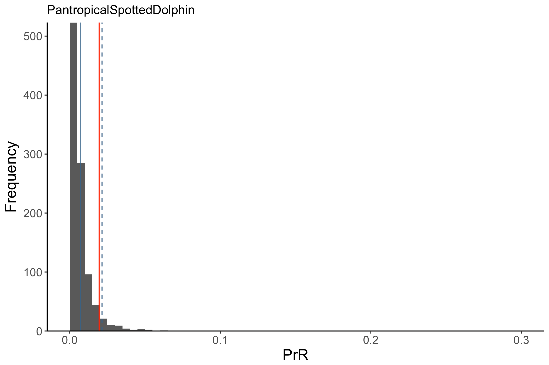

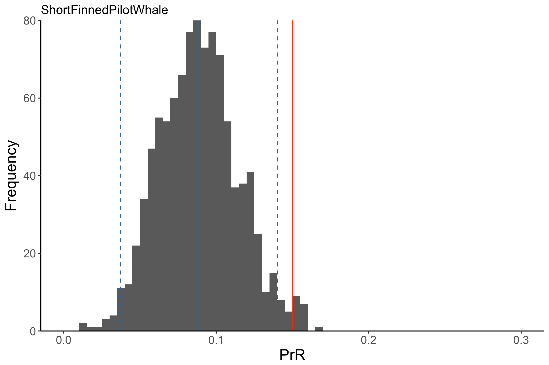

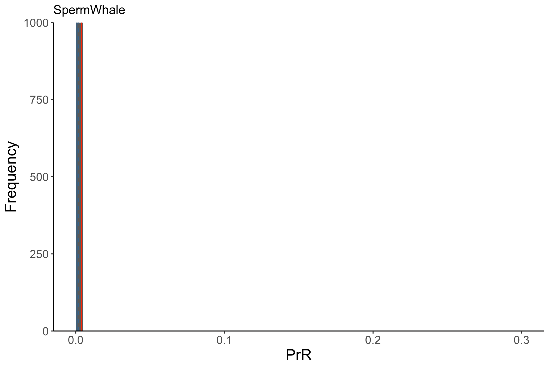


**Figure S3.3**

Phys-PrR calculated from 1000 populations with error in age estimation introduced within ten percent of the observed age of any given corpora count. For all figures, red line shows the observed Phy-PrR. Solid blue line is the mean of the error estimated Phys-PrRs, dashed blue lines show +/- two standard deviations of that mean. The PrRs of beluga whales, narwhals and short-finned pilot whales are consistently high with this form of age error.


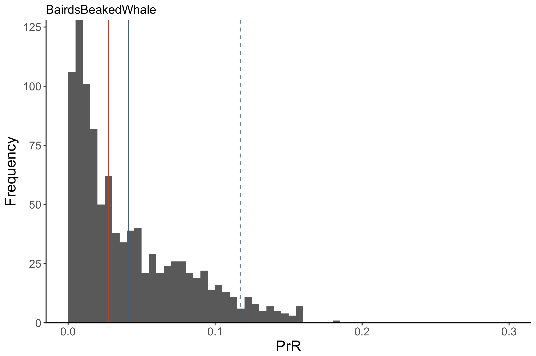

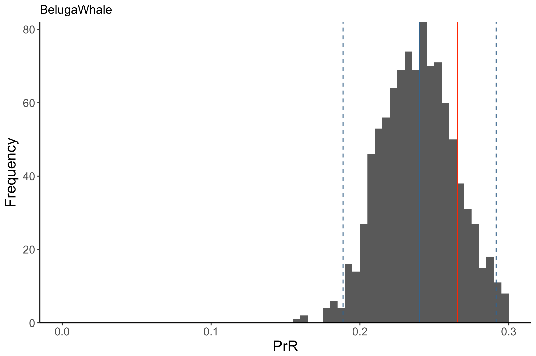

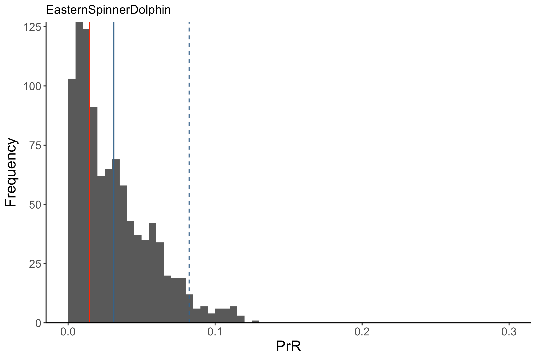

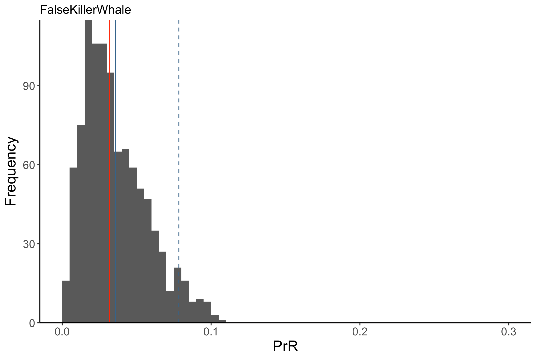

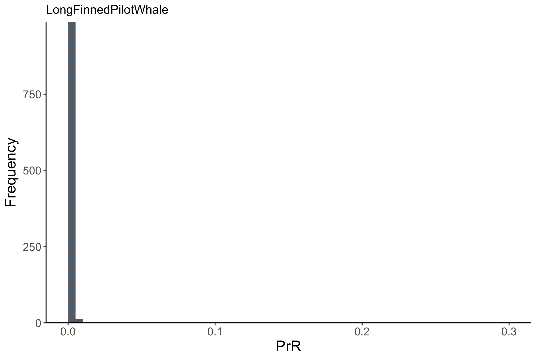

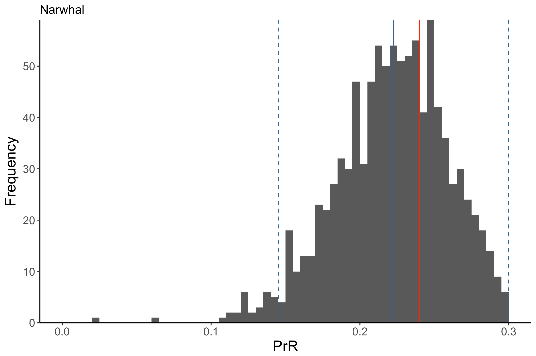

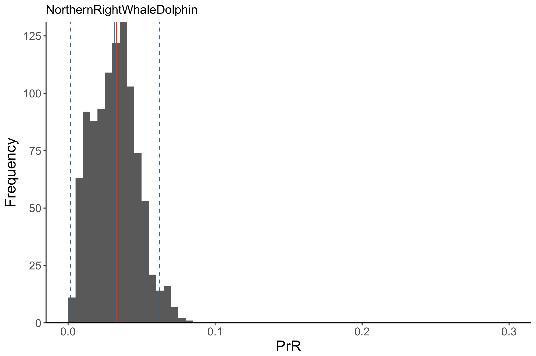

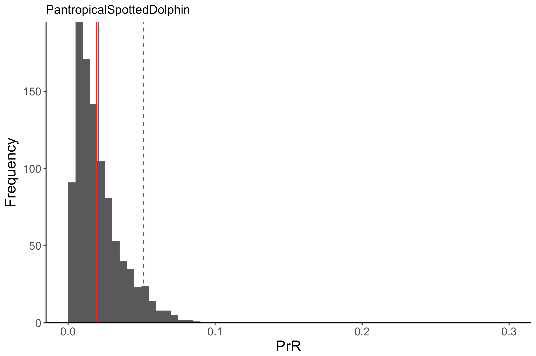

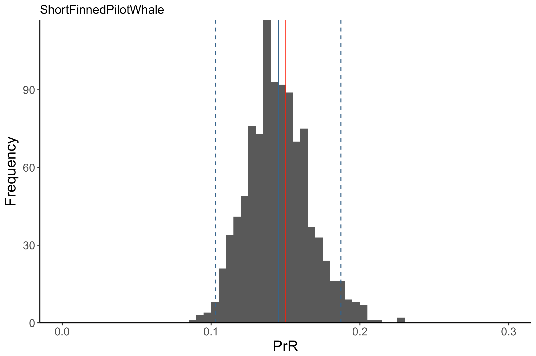

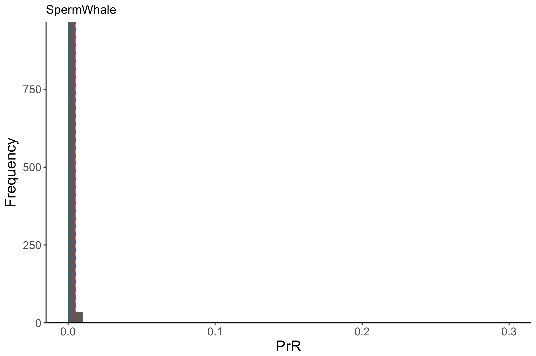


**References**

1. Sergeant, D. E., St. Aubin, D. J. & Geraci, J. R. Life history and northwest Atlantic status of Atlantic white-sided dolphin *Lagenorhynchus actus*. *Cetology* **30,** 1–12 (1980).

2. Kasuya, T., Brownell Jr, R. L. & Balcomb, K. C. Life history of Baird’s beaked whales. *Rep. Int. Whal. Comm.* **47,** 969–979 (1997).

3. Suydam, R. S. *Age, growth, reproduction, and movements of beluga whales (Delphinapterus leucas) from the eastern Chukchi Sea*. *PhD Thesis* (2009).

4. Ferreira, I. M., Kasuya, T., Marsh, H. & Best, P. B. False killer whales (*Pseudorca crassidens*) from Japan and South Africa: Differences in growth and reproduction. *Mar. Mammal Sci.* **30,** 64–84 (2014).

5. Murphy, S. *et al.* Reproductive failure in UK harbour porpoises *Phocoena phocoena*: Legacy of pollutant exposure? *PLoS One* **10,** 1–32 (2015).

6. Martin, A. R. & Rothery, P. Reproductive parameters of female long-finned pilot whales (*Globicephala melas*) around the Faroe Islands. *Rep. Int. Whal. Comm.* 263–304 (1993).

7. Amano, M. *et al.* Life history and group composition of melon-headed whales based on mass strandings in Japan. *Mar. Mammal Sci.* **30,** 480–493 (2014).

8. Ferrero, R. C. & Walker, W. A. Growth and reproduction of the northern right whale dolphin  *Lissodelphis borealis*  in the offshore waters of the North Pacific Ocean. *Can. J. Zool.* **71,** 2335–2344 (1993).

9. Kasuya, T. Reconsideration of life history parameters of the spotted and striped dolphin based on cemental layers. *Sci. Reports Whales Res. Institute, Tokyo* **28,** (1976).

10. Dabin, W., Cossais, F., Pierce, G. J. & Ridoux, V. Do ovarian scars persist with age in all Cetaceans: New insight from the short-beaked common dolphin (Delphinus delphis Linnaeus, 1758). *Mar. Biol.* **156,** 127–139 (2008).

11. Kasuya, T. & Marsh, H. Life history and reproductive biology of the short-finned pilot whale, *Globicephala macrorhynchus*, off the Pacific coast of Japan. *Rep. Int. Whal. Comm.* 259–310 (1984).

12. Ohsumi, S. Reproduction of the sperm whale in the north-west Pacific. *Sci. Reports Whales Res. Institute, Tokyo* **19,** 1–35 (1965).

13. Perrin, W. F., Holts, D. B. & Miller, R. B. Growth and reproduction of the eastern spinner dolphin, a geographical form of Stenella longirostris in the Eastern Tropical Pacific. *Fish. Bull.* **75,** 725–750 (1977).

14. Garde, E. *et al.* Life history parameters of narwhals (*Monodon monoceros*) from Greenland. *J. Mammal.* **96,** 866–879 (2015).

# Supplementary 4: Species used in Phylogenetic Comparative Analysis

Table S4. Species used in the phylogenetic ancestral state reconstruction analysis. A through literature search of all 72 toothed whale species revealed 12 other species where the lack of post-reproductive lifespan could be confirmed. The origin and nature of the data used are listed here.

| Species | Last Reproduction and Age Data | Details | Post-Reproductive LifeSpan ? | Refs |
| --- | --- | --- | --- | --- |
| Atlantic white-sided dolphin | Yes | Oldest female in sample (for chemical analysis) was lactating. (age 14- oldest in corpora sample approx. 13). | No | [1] |
| Baird’s beaked whale | This study | No reproductive senescence | No | [2] |
| Beluga | This study | PRLS | Yes | [3] |
| Common bottlenose dolphin | This study | No reproductive senescence | No | [4,5] |
| Dall’s porpoise | Yes | Oldest female sampled was pregnant | No | [6] |
| Dusky dolphin | Yes | Oldest observed female (aged 11) was reproductive | No | [7] |
| Dwarf sperm whale | Yes | Oldest observed female was pregnant | No | [8] |
| False killer whale | This study | No PRLS | No | [9] |
| Franciscana | Yes | Older individuals still observed as pregnant | No | [10] |
| Fraser’s dolphin | Yes | Oldest observed female was pregnant | No | [11] |
| Harbor porpoise | Yes | Oldest female in the sample was pregnant | No |  |
| Indo-Pacific finless porpoise | Yes | Oldest female sampled lactating. Second oldest pregnant | No | [12] |
| Killer whale | Yes | PRLS reported and tested. | Yes | [13–15] |
| Long-finned pilot whale | This study | No PRLS | No | [16] |
| Melon-headed whale | This study | No reproductive senescence | No | [17] |
| Narwhal | This study | PRLS | Yes | [18] |
| Northern right-whale dolphin | This study | No PRLS | No | [19] |
| Pacific white-sided dolphin | Yes | All mature females (10 individuals) were either pregnant or lactating. Oldest was 25+ which is higher by 10 years than the oldest atlantic white sided recorded | No | [20] |
| Pantropical spotted dolphin | This study | No PRLS | No | [21] |
| Pygmy sperm whale | Yes | Oldest female also the oldest pregnant female | No | [8] |
| short-beaked Common dolphin | Yes | Females in the oldest age category have a lower proportion pregnant but a higher proportion lactating | No | [22] |
| Short-finned pilot whale | This study | PRLS | Yes | [23] |
| Sperm whale | This study | No PRLS | No | [24] |
| Spinner dolphin | This study | No PRLS | No | [25] |
| Striped dolphin | This study | No reproductive senescence | No | [21] |

**References**

1. McKenzie C, Rogan E, Reid RJ, Wells DE. 1997 Concentrations and patterns of organic contaminants in Atlantic white-sided dolphins (*Lagenorhynchus acutus*) from Irish and Scottish coastal waters. *Environ. Pollut.* **98**, 15–27. (doi:10.1016/S0269-7491(97)00109-7)

2. Kasuya T, Brownell Jr RL, Balcomb KC. 1997 Life history of Baird’s beaked whales. *Rep. Int. Whal. Comm.* **47**, 969–979.

3. Suydam RS. 2009 *Age, growth, reproduction, and movements of beluga whales (Delphinapterus leucas) from the eastern Chukchi Sea*. PhD thesis: University of Washington.

4. Kasuya T, Izumisawa Y, Komyo Y, Ishino Y, Maejima Y. 1997 Life history parameters Bottlenose dolphins off Japan. *IBI Reports* **7**, 71–107.

5. Cockcroft VG, Ross GJB. 1990 Age, growth, and reproduction of Bottlenose dolphins *Tursiops truncatus* from the east coast of southern Africa. *Fish. Bull.* **88**, 289–302.

6. Kasuya T. 1978 Life History of Dall’s Porpoise with special refernce to the stock off the pacific coast of Japan. *Sci. Reports Whales Res. Institute, Tokyo* **30**, 1–63.

7. Dans SL, Crespo EA, Pedraza SN, Koen Alonso M. 1997 Notes on the reproductive biology of female dusky dolphins (*Lagenorhynchus obscurus*) off the Patagonian coast. *Mar. Mammal Sci.* **13**, 303–307. (doi:10.1111/j.1748-7692.1997.tb00633.x)

8. Plön S. 2004 The status and natural history of pygmy (Kogia breviceps) and dwarf (K . sima) sperm whales off Southern Africa.

9. Ferreira IM, Kasuya T, Marsh H, Best PB. 2014 False killer whales (*Pseudorca crassidens*) from Japan and South Africa: Differences in growth and reproduction. *Mar. Mammal Sci.* **30**, 64–84. (doi:10.1111/mms.12021)

10. Danilewicz D. 2003 Reproduction of female franciscana (*Pontoporia blainvillei*) in Rio Grande do Sul, Southern Brazil. *Lat. Am. J. Aquat. Mamm.* **2**, 67–78. (doi:10.5597/lajam00034)

11. Amano M, Miyazaki N, Yanagisawa F. 1996 Life history of Fraser’s dolphin Lagenodelphis hosei , based on a school captured off the pacific coast of Japan. *Mar. Mammal Sci.* **12**, 199–214.

12. Shirakihara M, Takemura A, Shirakihara K. 1993 Age, growth and reproduction of the finless porpoise, Neophocaena phocaenoides in the coastal waters of western Kyushu, Japan. *Mar. Mammal Sci.* **9**, 41–46.

13. Olesiuk PF, Ellis GM, Ford JKB. 2005 Life History and Population Dynamics of Northern Resident Killer Whales (Orcinus orca) in British Columbia.

14. Foote AD. 2008 Mortality rate acceleration and post-reproductive lifespan in matrilineal whale species. *Biol. Lett.* **4**, 189–91. (doi:10.1098/rsbl.2008.0006)

15. Croft DP, Brent LJN, Franks DW, Cant MA. 2015 The evolution of prolonged life after reproduction. *Trends Ecol. Evol.* **30**, 407–416. (doi:10.1016/j.tree.2015.04.011)

16. Martin AR, Rothery P. 1993 Reproductive parameters of female long-finned pilot whales (*Globicephala melas*) around the Faroe Islands. *Rep. Int. Whal. Comm.* , 263–304.

17. Amano M, Yamada TK, Kuramochi T, Hayano A, Kazumi A, Sakai T. 2014 Life history and group composition of melon-headed whales based on mass strandings in Japan. *Mar. Mammal Sci.* **30**, 480–493. (doi:10.1111/mms.12050)

18. Garde E, Hansen SH, Ditlevsen S, Tvermosegaard KB, Hansen J, Harding KC, Heide-Jørgensen MP. 2015 Life history parameters of narwhals (*Monodon monoceros*) from Greenland. *J. Mammal.* **96**, 866–879. (doi:10.1093/jmammal/gyv110)

19. Ferrero RC, Walker WA. 1993 Growth and reproduction of the northern right whale dolphin  *Lissodelphis borealis*  in the offshore waters of the North Pacific Ocean. *Can. J. Zool.* **71**, 2335–2344.

20. Ferrero RC, Walker WA. 1994 Age, growth, and reproductive patterns of the Pacific white-sided dolphin (*Lagenorhynchus obliquidens*) taken in high seas drift nets in the central North Pacific Ocean. *Can. J. Zool.* **74**, 1673–1687. (doi:10.1139/z96-185)

21. Kasuya T. 1976 Reconsideration of life history parameters of the spotted and striped dolphin based on cemental layers. *Sci. Reports Whales Res. Institute, Tokyo* **28**.

22. Danil K, Chilvers S, Ellis H, Perrin WF, Ripley BJ. 2004 Growth and reproduction of female short-beaked common dolphins, Delphinus delphis, in the eastern tropical Pacific. *Mar. Sci.* , 192. (doi:10.1139/z06-188)

23. Dabin W, Cossais F, Pierce GJ, Ridoux V. 2008 Do ovarian scars persist with age in all Cetaceans: New insight from the short-beaked common dolphin (Delphinus delphis Linnaeus, 1758). *Mar. Biol.* **156**, 127–139. (doi:10.1007/s00227-008-1070-4)

24. Ohsumi S. 1965 Reproduction of the sperm whale in the north-west Pacific. *Sci. Reports Whales Res. Institute, Tokyo* **19**, 1–35.

25. Perrin WF, Holts DB, Miller RB. 1977 Growth and reproduction of the eastern spinner dolphin, a geographical form of Stenella longirostris in the Eastern Tropical Pacific. *Fish. Bull.* **75**, 725–750.

# Supplementary 5: Additional Species Data

Additional biological information about the species used in this study. Additional fecundity data describes any extra data describing how reproductive effort changes with age in these species

| Species | General | Additional Fecundity Data | Social Data |
| --- | --- | --- | --- |
| Atlantic white-sided dolphin  *Lagenorhynchus acutus* | Oceanic dolphin distributed in the North Atlantic. Do not appear to be deep divers, most feeding is on fish and squid near the surface [1]. | Oldest female (aged 14) in dolphins stranded around the coast of the British Isles was lactating [2]. Oldest individual in corpora data is 17 [3] No clear accounts of pregnancy but ‘most’ females appear to be either pregnant of lactating [1]. | Appear to be transitory over large areas. Mixed sex groups of very variable size. In the western Atlantic mean group size is 71 in late-spring but 35 in late summer [4]. |
| Baird’s beaked whale  *Berardius bairdii* | Distributed in temperate water in the North Pacific mainly in the deep waters over the continental slope [5]. Deep divers feeding mainly on benthic fish and cephalopods [6]. Males live up to 84 whereas females live up to 59 [6]. | There is no apparent decline in pregnancy rate with age, and one of three females in the oldest age bracket (50-59) was lactating [7]. Previous studies have reproted no evidence of a female post-reproductive life span in this species [6]. | Usually found in schools, consisting of up to 30 whales. No evidence of sexual segregation. 14% are sighted alone [6]. |
| Beluga whale  *Delphinapterus leucas* | Beluga inhabit the polar and sub-polar waters in the northern hemisphere with the exception of the Atlantic section of the Arctic ocean[8,9]. Beluga whales breed in summer in coastal areas and then winter around the edge of the pack ice further out to sea [10]. At least 19 populations (or stocks) are present worldwide, defined by their summer wintering grounds [11]. Diet is varied and seasonal including both fish and invertebrate prey [12]. | The production of calves appears to decrease with age in female beluga whales. Pregnancy rates remain high until around 25 and then decline in older animas [13]. A similar pattern of reduced pregnancy in older females was found in a separate study of Beluga whales- though difficulties with ageing precludes accurately assigning age [14]. In addition no females over the age of 41 had any corpus lutenum, which would suggest a recent ovulation [13]. A similar pattern of slowing corpora deposition in old age was found in a separate Beluga sample [15]. | Beluga whales show high fidelity to their summering grounds, which appear to be learnt from their mothers evidenced by strong mtDNA differentiation between summering grounds [16–18]- this pattern is maintained at local scales within feeding grounds [19]. Recent evidence suggests these populations are also be spatially separated during the winter [10]. Genetic evidence suggests that adult females continue to associate with their mothers, at least during the summer migrations [20] This is also supported by behavioural observations [21]. Adult males are usually found in single sex male-alliances but are still usually found in areas similar to their female relatives [20]. |
| Common bottlenose dolphin  *Tursiops truncatus*  (3 populations) | Worldwide distribution. Most commonly in coastal waters in tropical and temperate regions, but are also found in pelagic waters. Very variable diet and ranging patterns [22,23]. | 48 year old females *T. trucatus* have be observed to successfully give birth. Females appear to ovulate repeatedly during a given breeding season [24]. Some evidence suggests a moderate decline in pregnancy rate with age, from 50% age 10 to 34% between at 30-34 [25]. But pregnant and lactating females appear to be present at all ages [25]. | Bottlenose dolphins have a varied and complex social system. Well-studied resident populations in Shark Bay Australia and Sarasota Bay USA are characterised by sex segregated societies. Females have a large and network of associates based around a core ‘band’. Males for complex multi-level alliances to dominate access to females [23]. |
| False killer whale  *Pseudorca crassidens* | Distributed widely in temperate and tropical waters. Mostly pelagic but occasionally observed in-shore. False killer whales feed on a very diverse sources, generally fish and squid but they have been observed attacking other whale species [26]. | Previous studies have suggested an observed decline in ovulation rate with age in false killer whales may lead to a post-reproductive lifespan [27,28]. No pregnancy were observed among 13 *P. crassidens* greater than 44 years old (max age 55) [27]. Detailed examination of the corpora has concluded that corpora albanica are not reabsorbed with age in false killer whales [28]. | High incidence of mass stranding’s suggests that they are highly social [26]. Stranded groups are of mixed sex but usually contain more females than males [28]. Limited photo-identification of false killer whales off Costa Rica has shown the same whales being observed together after an 11 month interval suggesting a stable pattern of associates [29]. |
| Harbour porpoise  *Phocoena phocoena* | Widely distributed in sub-polar and temperate coastal waters in the Northern Hemisphere [30]. Harbour porpoises feed at a very high rate on small fish [31]. | The oldest individuals in the harbour porpoise sample used in this study were both pregnant [32]. In addition in a separate study, the oldest individual (aged 17) in a sample of 239 porpoises had recently given birth[33]. Although some females in this sample were in their late-teens the majority were younger than 12 [33]. | Harbour porpoises are usually observed in groups of 1-3 individuals and sometimes in larger aggregations of 6-8 individuals [30]. |
| Long-finned pilot whale  *Globicephala melas* | Distributed in temperate and sub-polar waters in the north and south Atlantic and the south Pacific [34]. Long-finned pilot whales are deep divers feeding mainly on squid, but also eating a variety of fish and crustaceans [35]. | Reduced ovulation rate and pregnancy rate has been observed older long-finned pilot whales [36]. A regression of pregnancy rate and age suggested that females should pregnancy will halt at 55-56 years of age, after which there are only one or two females alive, which compares favourably to the predicted age at which we predict 95% of lifetime fecundity will be completed (age 54). In addition, one of the three oldest females in a sample of 1443 long-finned pilot whales was pregnant [36]. | Long-finned pilot whales are extremely social, as evidenced by the very high incidence of mass-strandings [34]. Genetic evidence points to a hierarchical social structure based around male and female philopatry and out-group mating [37]. |
| Melon-headed whale  *Peponocephala electra* | *P. electra* is a primarily pelagic species found worldwide in tropical and sub-tropical waters. Occasionally found in-shore but mainly associated offshore deep waters [38]. Feeding ecology is not well-studied but appears to consist mainly of pelagic fish and squid [39] | Annual pregnancy rates match annual ovulation rates (based on corpora counts) in *P. electra*, suggesting corpora counts a re a good measure of pregnancy rate[40]. Their patterns of maturation (at around 7) and inter-birth intervals (short and regular) have more in common with small dolphin species than with the most closely related short-finned pilot whales and killer whales [40]. | Large mass stranding’s suggest that melon-headed whales are social [38,40]. Observations of whales off Hawaii suggest that variable group sizes from single individuals to groups of over 800 individuals, with a median group size of 275 [41]. There is some evidence of groups of mature males moving between groups, perhaps suggesting male-biased dispersal and female philopatry [40]. |
| Narwhal  *Monodon monoceros* | Narwhals live in polar and sub-polar waters of the Atlantic sector of the Arctic Ocean [42]. They form distinct populations concentrated around west Greenland, Baffin Bay and northern Hudson Bay [43]. Like Beluga whales, Narwhals spend the summer in coastal waters and then the winter is spent further out over the continental shelf among the pack ice in the winter [44,45]. Diet varies between populations and sexes but consist mainly of pelagic and benthic fish and squid [46,47] | Previous authors have suggested that some females may be post-reproductive in this species [48]. Limited pregnancy available for from the study used to provide corpora data. Only one female over the after of the age of 47 was found to be reproductively active [48]. That one exception is pregnant female of 69 [48]. Interestingly there is a single 69 year old female with an exceptionally high corpora count (24 corpora, whereas other females of a similar age have between 4-10[48]). This may suggest a mixed strategy in Narwhals with most females ceasing reproduction in their late 40s but some continuing to reproduce until much later. It could also suggest a recording or ageing error during data collection. Interpretation of older records of reproductive history in Narwhal is hindered by the difficulty in ageing Narwhal using dental layers [49]. | Narwhal social structures are largely unknown. Observations of migrating whales has suggested that they move in small clusters consisting of, on average, 3-4 individuals (range 1-25) [50]. As many as 642 clusters can appear to move together as part of a large ‘herd’ [50]. The clusters themselves are single sex, either of mothers with young or males [50]. Due to the conserved summering grounds it is thought that the species may be at least matrifocal, with young learning their migration routes from their mother [51] |
| Northern right-whale dolphin  *Lissodelphis borealis* | *L. borealis* inhabits sub-Arctic waters in the northern pacific. Their main prey appears to be squid and lantern fish [52]. | Previous examination of the corpora count data has also suggested that ovarian activity slows in later life (after age 21) [53]. | Very little is known about the social system of the northern right whale dolphins. They are often found in interspecies associations, having been observed with 14 other species [52]. The closely related southern right-whale dolphin (*Lissodelphis peroneii*) of the south Pacific is usually observed in large groups of greater than 200 [54]. |
| Pantropical spotted dolphin  *Stenella attenuata* | *S. attenuate*  are oceanic dolphin with a worldwide distribution in tropical and occasionally subtropical waters [55]. Prey is dominated by mesopelagic fish and squid.[56]. | The pregnancy rate of females in the oldest age category (35-46) is the second highest of any age category, and higher than the population pregnancy rate [57]. | In common with other oceanic dolphins, *S attenuata*  is usually observed in very large associations, often these associations are mixed species including other dolphin species and tuna [55,58]. There do seem to be lower levels of organisation within these large schools, with a close association between mothers and young, perhaps lasting until they are juvenile [59]. Adult males appear to form distinct subgroups within the larger school [59]. |
| Short-finned pilot whale  *Globicephala macrorhynchus* | Short-finned pilot whales inhabit topical and sub-tropical waters worldwide [34]. short-finned pilot whales perform deep dives hunting primarily squid at depths of 1019m [60,61]. | No pregnant *G. macrorhynchus* females over 39 years of age, despite a maximum survival to 63 [62]. Lactation seems to last much longer than pregnancy and ovulation [62]. For example a female of 48.5 who had not recently ovulated was still lactating [63]. Detailed examination of the corpora in short-finned pilot whales has led to the conclusion that corpora do not regress with age [63]. | Mass stranding’s of short-finned pilot whales suggest that they are highly social and live in mixed sex groups [62]. Observation of photo-identified whales has also suggested that they live in permeant mixed sex groups with males mating outside the group [64]. This suggests a social system based on matrilineal philopatry and out-group mating like killer whales and long-finned pilot whales [37,65]. |
| Short-beaked common dolphin  *Delphinus delphis* | *D. delphis* has a worldwide predominantly coastal distribution concentrated in temperate waters with some populations ins sub-tropical regions [66]. They have a variable diet but mainly small mesopelagic and epipelagic fish and squid [66,67]. | The oldest *D. delphis* female in a western north Atlantic populations was over 30 years old (older than any samples in this study) and was also pregnant [68]. In a sample from the eastern tropical Pacific there was no evidence of reproductive senescence in any of the 440 females studied [69]. | Sex ratio of dolphins caught in nets suggest sexual segregation in adult short-beaked common dolphins [68] |
| Sperm Whale  *Physeter macrocephalus* | Sperm whales have a worldwide distribution. Females and calves in habit deep tropical and subtropical waters less the latitude 40° (50° in the North Pacific). Mature males inhabit the polar and sub-polar waters travelling to the tropics to breed and then returning to the poles [70]. | There is mixed evidence for a post-reproductive lifespan in sperm whales. In a large scale study of sperm whales caught off South Africa no whales older than 41 were pregnant, but whales of 48 were lactating [71]. However, in whales caught off Japan (used in this study) females up to 60 were found to be pregnant or recently pregnant (evidenced by a recent corpus lutenum) [72]. Corpora do not appear to regress in Sperm Whales, multiple ovulations also appear to be rare [72]. It was also found the whales with a high number of corpora were much less commonly pregnant than those with a low number of corpora, which may be indicative of older females who have ovulated many times being less likely to be pregnant than younger females who have ovulated less often [72]. | Female sperm whales have hierarchically clustered societies. Whales have networks of a selection of preferential associates called a ‘unit’. These units themselves preferentially associate into clans, usually determined on the basis of shared vocalisations [73–75]. These social units usually consist of several matrilines [70]. |
| Spinner Dolphin  Stenella longirostris | Wide distribution in all tropical and most sub-tropical waters worldwide [76]. Usually pelagic but with some costal populations [76]. Diet consist mainly of mesopelagic fish, with some cephalopods [77] | Pregnancy rates of spinner dolphins decrease with age: approx. 35% of females between 9 and 14 pregnant whereas only around 20% of females between 14 and 22 are pregnant [78]. Four of the 536 females examined for the corpora data used in this study were neither pregnant or lactating, had a high number of corpora and had small withered ovaries and have no developing follicles [78]. This could mean that these females were post-reproductive [78], if this is the case it is very rare and unlikely to be evolutionary significant. | Spinner Dolphins appear to live in very variable group sizes, usually around 120 individuals but have been observed in groups of 2600 [79,80]. Social structure varies by region and may be linked to residency patterns and local ecology [80]. Dispersal does not appear to be male-biased, with both males and females having an equal likelihood of dispersing [80]. |
| Striped Dolphin  *Stenella coeruleoalba* | Pelagic dolphin common in warm-temperate and tropical waters worldwide [81,82]. Diet varies regionally but is generally a mixture of pelagic and benthopelagic cephalopods and fish [82]. | The proportion of females aged 35-49 pregnant is lower than the proportion pregnant in other age categories (50% vs 65.1-77.3%). However there are very few females who survive to this age which is likely to make the estimates unreliable [57]. | Striped dolphins form schools of variable both within and between regions, from 10 individuals to over 500 [82]. In the western pacific schools exclusively of adults and juveniles are found as well as mixed schools [83]. |

**References**

1. Cipriano F. 2002 Atlantic White-Sided Dolphin  *Lagenorhynchus actus* . In *Encyclopedia of Marine Mammals* (eds WF Perrin, B Würsig, JGM Thewissen), pp. 49–51. London: Academic Press.

2. McKenzie C, Rogan E, Reid RJ, Wells DE. 1997 Concentrations and patterns of organic contaminants in Atlantic white-sided dolphins (*Lagenorhynchus acutus*) from Irish and Scottish coastal waters. *Environ. Pollut.* **98**, 15–27. (doi:10.1016/S0269-7491(97)00109-7)

3. Sergeant DE, St. Aubin DJ, Geraci JR. 1980 Life history and northwest Atlantic status of Atlantic white-sided dolphin *Lagenorhynchus actus*. *Cetology* **30**, 1–12.

4. Weinrich MT, Belt C, Morin D. 2001 Behavior and Ecology of the Atlantic White-Sided Dolphin (*Lagenorhynchus acutus*) in Coastal New England Waters. *Mar. Mammal Sci.* **17**, 231–248.

5. MacLeod CD *et al.* 2006 Known and inferred distributions of beaked whale species ( Cetacea : Ziphiidae ). *J. Cetecean Res. Manag.* **7**, 271–286.

6. Kasuya T. 2002 Giant Beaked Whales *Berardius bairdii* and *B. arnuxii*. In *Encyclopedia of Marine Mammals* (eds WF Perrin, B Würsig, JGM Thewissen), pp. 519–523. London: Academic Press.

7. Kasuya T, Brownell Jr RL, Balcomb KC. 1997 Life history of Baird’s beaked whales. *Rep. Int. Whal. Comm.* **47**, 969–979.

8. Brodie PF. 1989 The White Whale Delphinapterus leucas (Pallas, 1776). In *Handbook of Marine Mammals (book 4)* (eds SH Ridgway, R Harrison), pp. 119–144. Academic Press.

9. O’Corry-Crowe G. 2002 Beluga Whale *Delphinapterus leucas* . In *Encyclopedia of Marine Mammals* (eds WF Perrin, B Würsig, JGM Thewissen), pp. 94–99. London: Academic Press.

10. Citta JJ *et al.* 2016 Satellite telemetry reveals population specific winter ranges of beluga whales in the Bering Sea. *Mar. Mammal Sci.* (doi:10.1111/mms.12357)

11. Laidre KL *et al.* 2015 Arctic marine mammal population status , sea ice habitat loss , and conservation recommendations for the 21st century. *Conserv. Biol.* **29**, 724–737. (doi:10.1111/cobi.12474)

12. Seaman GA, Lowry LF, Frost KJ. 1982 Foods of belukha whales (*Delphina[terus leucas*) in Western Alaska. *Cetology* **44**, 1–19.

13. Suydam RS. 2009 *Age, growth, reproduction, and movements of beluga whales (Delphinapterus leucas) from the eastern Chukchi Sea*. PhD thesis: University of Washington.

14. Burns JJ, Seaman GA. 1986 Investigations of Belukha Whales n Costal Waters of Western and Northern Alaska: II. Biology and Ecology.

15. Harwood LA, Kingsley MCS, Pokiak F. 2015 Monitoring beluga harvests in the Mackenzie delta and near Paulatuk, NT ,Canada : harvest efficiency and trend, size and sex of landed whales, and reproduction, 1970- 2009. *Can. Mauscript Rep. Fish. Aquat. Sci. 3059*

16. Turgeon J, Duschesne P, Colbeck GJ, Postma LD, Hammill MO. 2011 Spatiotemporal segregation among summer stocks of beluga (*Delphinapterus leucas*) despite nuclear gene flow : Implication for the endangered belugas in Eastern Hudson Bay. *Conserv. Genet.* **13**, 419–433. (doi:10.1007/s10592-011-0294-x)

17. Meschersky IG, Shpak O V, Litovka DI, Glazov DM, Borisova EA, Rozhnov V V. 2013 A Genetic Analysis of the Beluga Whale *Delphinapterus leucas* (Cetacea : Monodontidae) from Summer Aggregations in the Russian Far East. *Russ. J. Mar. Biol.* **39**, 125–135. (doi:10.1134/S1063074013020065)

18. Palsbøll PJ, Heide-Jørgensen MP, M B. 2002 Analysis of mitochondiral control refion nucleotide sequences from Baffin Bay beluga, (*Delphinapterus leucas*): detecting pods or sub-populations? *NAMMCO Sci. Publ. Vol. 4* **Belugas in**, 39–50.

19. Corry-crowe GO *et al.* 2018 *Migratory culture , population structure and stock identity in North Pacific beluga whales ( Delphinapterus leucas )*. (doi:10.5061/dryad.6b70g11.Microsatellite)

20. Colbeck GJ, Duchesne P, Postma LD, Lesage V, Hammill MO, Turgeon J. 2013 Groups of related belugas (*Delphinapterus leucas*) travel together during their seasonal migrations in and around Hudson Bay. *Proc. R. Soc. B* **280**, 20122552. (doi:10.1098/rspb.2012.2552)

21. Smith TG, Hammill MO, Martin AR. 1994 Herd composition and behaviour of white whales (Delphinapterus leucas) in two Candian arctic esturies. *Bioscience* **39**, 1994.

22. Wells RS, Scott MD. 2002 Bottlenose Dolphins  *Tursiops truncatus*  and  *T. aduncus* . In *Encyclopedia of Marine Mammals* (eds WF Perrin, B Würsig, JGM Thewissen), pp. 122–128. London: Academic Press.

23. Connor RC, Randall, S W, Mann J, Read AJ. 2000 The bottlenose dolphin: social relationships in a fission fusion society. In *Cetecean Societies: Field Studies of Dolphins and Whales* (eds J Mann, RC Connor, PL Tyack, H Whitehead), pp. 91–127. London: The Univerisity of Chicago Press Ltd.

24. Wells RS, Scott MD. 1999 Bottlenose dolphin  *Tursops truncatus*  (Montagu, 1821). In *Handbook of Marine Mammals Vol 6: the second book of Dolphins and Porpoises* (eds SH Ridgeway, R Harrison), pp. 137–182. San Diego: Academic Press.

25. Marsh H, Kasuya T. 1986 Evidence for reproductive senescence in female ceaceans. *Rep. Int. Whal. Comm.* **Special Is**, 57–74.

26. Baird RW. 2002 False Killer Whale *Pseudorca crassidens* . In *Encyclopedia of Marine Mammals* (eds WF Perrin, B Würsig, JGM Thewissen), pp. 411–412. London: Academic Press.

27. Ferreira IM. 2008 Growth and reproduction in false killer whales (*Pseudorca crassidens* Owens, 1846).

28. Ferreira IM, Kasuya T, Marsh H, Best PB. 2014 False killer whales (*Pseudorca crassidens*) from Japan and South Africa: Differences in growth and reproduction. *Mar. Mammal Sci.* **30**, 64–84. (doi:10.1111/mms.12021)

29. Alejandro A-G, Brennan B, Rodriguez P, Thomas M. 1997 Resightings and behavior of False Killer Whales (*Pseudorca crassidens*) in Costa Rica. *Mar. Mammal Sci.* **13**, 307–314.

30. Bjørge B, Tolley KA. 2002 Harbor Porpoise *Phocoena phocoena* . In *Encyclopedia of Marine Mammals* (eds WF Perrin, B Würsig, JGM Thewissen), pp. 549–551. London: Academic Press.

31. Wisniewska DM, Johnson M, Teilmann J, Rojano-Donate L, Shearer J, Sveegard S, Miller LA, Siebert U, Madsen PT. 2016 Ultra-High Foraging Rates of Harbor Porpoises Make Them Vulnerable to Anthropogenic Disturbance Report Ultra-High Foraging Rates of Harbor Porpoises Make Them Vulnerable to Anthropogenic Disturbance. *Curr. Biol.* **26**, 1441–1446. (doi:10.1016/j.cub.2016.03.069)

32. Murphy S *et al.* 2015 Reproductive failure in UK harbour porpoises *Phocoena phocoena*: Legacy of pollutant exposure? *PLoS One* **10**, 1–32. (doi:10.1371/journal.pone.0131085)

33. Read AJ, Hohn A a. 1995 Life in the fast lane: the life history of harbor porpoises from the Gulf of Maine. *Mar. Mammal Sci.* **11**, 423–440. (doi:10.1111/j.1748-7692.1995.tb00667.x)

34. Olson PA, Reilly SB. 2002 Pilot Whales  *Globicephala melas*  and  *G. macrorhynchus* . In *Encyclopedia of Marine Mammals* (eds WF Perrin, B Würsig, JGM Thewissen), pp. 898–903. London: Academic Press.

35. Desportes G, Mouritsen R. 1993 Preliminary Results on the Diet of Long-Finned Pilot Whales off the Faroe Islands. *Rep. Int. Whal. Comm. Spec. Issue 4* **SI 4**, 305–324.

36. Martin AR, Rothery P. 1993 Reproductive parameters of female long-finned pilot whales (*Globicephala melas*) around the Faroe Islands. *Rep. Int. Whal. Comm.* , 263–304.

37. Amos B, Schlötterer C, Tautz D. 1993 Social structure of pilot whales revealed by analytical DNA profiling. *Science (80-. ).* **260**, 670–672. (doi:10.1126/science.8480176)

38. Perryman WL. 2002 Melon-Headed Whale *Peponocephala electra*. In *Encyclopedia of Marine Mammals* (eds WF Perrin, B Würsig, JGM Thewissen), pp. 733–735. London: Academic Press.

39. Jefferson TA, Barros NB. 1997 *Peponocephala electra*. *Mamm. Species* **553**.

40. Amano M, Yamada TK, Kuramochi T, Hayano A, Kazumi A, Sakai T. 2014 Life history and group composition of melon-headed whales based on mass strandings in Japan. *Mar. Mammal Sci.* **30**, 480–493. (doi:10.1111/mms.12050)

41. Aschettino JM, Baird RW, McSweeney DL, Webster DL, Schorr GS, Huggins JL, Martinen KK, Mahaffy SD, West KL. 2012 Population structure of melon-headed whales (*Peponocephala electra*) in the Hawaiian Archipelago : Evidence of multiple populations based on photo identification. *Mar. Mammal Sci.* **28**, 666–689. (doi:10.1111/j.1748-7692.2011.00517.x)

42. Heide-Jørgensen MP. 2002 Narwhal *Monodon monoceros* . In *Encyclopedia of Marine Mammals* (eds WF Perrin, B Würsig, JGM Thewissen), pp. 783–787. London: Academic Press.

43. Petersen SD, Tenkula D, Ferguson SH. 2011 Population Genetic Structure of Narwhal (*Monodon monoceros*). *DFO Can. Sci. Advis. Secr. Res. Doc. 2011/021* **vi**.

44. Dietz R, Heide-Jørgensen MP. 1995 Movements and swimming speed of narwhals, Monodon monoceros, equipped with satellite transmitters in Melville Bay, Northwest Greenland. *Candian J. Zool.* **73**, 2106–2119. (doi:10.1139/z95-248)

45. Heide-Jørgensen MP, Dietz R, Laidre KL, Richard P, Orr J, Schmidt HC. 2003 The migratory behaviour of narwhals (*Monodon monoceros*). *Can. J. Zool.* **81**, 1298–1305. (doi:10.1139/Z03-117)

46. Watt CA, Heide-Jørgensen MP, Ferguson SH. 2013 How adaptable are narwhal? A comparison of foraging patterns among the world’s three narwhal populations. *Ecosphere* **4**, 1–15. (doi:10.1890/ES13-00137.1)

47. Watt CA, Orr JR, Nielsen NH, Ferguson SH. 2015 Differences in dive behaviour among the world’ s three narwhal *Monodon monoceros* populations correspond with dietary differences. *Mar. Ecol. Prog. Ser.* **525**, 273–285. (doi:10.3354/meps11202)

48. Garde E, Hansen SH, Ditlevsen S, Tvermosegaard KB, Hansen J, Harding KC, Heide-Jørgensen MP. 2015 Life history parameters of narwhals (*Monodon monoceros*) from Greenland. *J. Mammal.* **96**, 866–879. (doi:10.1093/jmammal/gyv110)

49. Garde E, Heide-jørgensen MP, Ditlevsen S, Hansen SH, Copenhagen Ø. 2012 Aspartic acid racemization rate in narwhal (*Monodon monoceros*) eye lens nuclei estimated by counting of growth layers in tusks. *Polar Res.* **1**, 1–7.

50. Marcoux M, Auger-Méthé M, Humphries MM. 2009 Encounter frequencies and grouping patterns of narwhals in Koluktoo Bay, Baffin Island. *Polar Biol.* **32**, 1705–1716. (doi:10.1007/s00300-009-0670-x)

51. Palsbøll PJ, Heide-Jørgensen MP, Dietz R. 1997 Population structure and seasonal movements of narwhals, *Monodon monoceros* , determined from mtDNA analysis. *Heredity (Edinb).* **78**, 284–292.

52. Lipsky JD. 2002 Right Whale Dolphins. In *Encyclopedia of Marine Mammals* (eds WF Perrin, B Würsig, JGM Thewissen), pp. 1030–1033. London: Academic Press.

53. Ferrero RC, Walker WA. 1993 Growth and reproduction of the northern right whale dolphin  *Lissodelphis borealis*  in the offshore waters of the North Pacific Ocean. *Can. J. Zool.* **71**, 2335–2344.

54. Rose B, Payne AIL. 1991 Occurrence and Behavior of the Southern Right Whale Dolphin Lissodelphis peronii off Namibia. *Mar. Mammal Sci.* **7**, 25–34. (doi:10.1111/j.1748-7692.1991.tb00547.x)

55. Perrin WF. 2002 Pantropical Spotted Dolphin *Stenella attenuata*. In *Encyclopedia of Marine Mammals* (eds WF Perrin, B Würsig, JGM Thewissen), pp. 865–867. London.

56. Robertson KM, Chivers SJ. 1997 Prey occurrence in pantropical spotted dolphins , *Stenella attenuata*, from the eastern tropical Pacific. *Fish. Bull.* **95**, 334–348.

57. Kasuya T. 1976 Reconsideration of life history parameters of the spotted and striped dolphin based on cemental layers. *Sci. Reports Whales Res. Institute, Tokyo* **28**.

58. Gowans S, Würsig B, Karczmarski L. 2007 The social structure and strategies of delphinids: predictions based on an ecological framework. *Adv. Mar. Biol.* **53**, 195–294. (doi:10.1016/S0065-2881(07)53003-8)

59. Pryor K, Sballenberger IK. 1991 Social structure in spotted dolphins (*Stenella attenuata*) in the tuna purse seine fishery in the Eastern Tropical Pacific. In *Dolphin Societies: Discoveries and Puzzles* (eds K Pryor, KS Norris), pp. 160–196. Berkley: University of California Press.

60. Hernández V, Martin V. 1994 Stomach contents of two short-finned pilot whale (Globicephala macrorhynchus Gray, 1846) (Cetecea, Delphinidae) off the Canary Islands: a preliminary note. *Int. Counc. Explor. Sea*.

61. Soto NA, Mark P, Madsen PT, Díaz F, Domínguez I, Brito A, Tyack PL. 2008 Cheetahs of the deep sea: deep foraging sprints in short-finned pilot whales off Tenerife (Canary Islands). *J. Anim. Ecol.* **77**, 936–947. (doi:10.1111/j.1365-2656.2008.01393.x)

62. Kasuya T, Marsh H. 1984 Life history and reproductive biology of the short-finned pilot whale, *Globicephala macrorhynchus*, off the Pacific coast of Japan. *Rep. Int. Whal. Comm.* , 259–310.

63. Marsh H, Kasuya T. 1984 Changes in the ovaries of the short-finned pilot whale, *Globicephala macrorhynchus*, with age and reproductive activity. *Rep. Int. Whal. Comm.* **6**, 311–335.

64. Heimlich-Boran JR. 1993 Social organisation of the short-finned pilot whale, *Globicephala macrorhynchus* , with special reference to the comparative social ecology of delphinids. *PhD Thesis*.

65. Bigg MA, Olesiuk PF, Ellis GM, Ford JKB, Balcomb KC. 1990 Social organization and genealogy of resident killer whales (*Orcinus orca*) in the coastal waters of British Columbia and Washington State. *Reports Int. Whal. Comm.* **SI 12**, 383–405.

66. Perrin WF. 2002 Common Dolphins  *Delphinus delphis* , *D. capensis*  and  *D. tropicalis* . In *Encyclopedia of Marine Mammals* (eds WF Perrin, B Würsig, JGM Thewussen), pp. 245–248. London: Academic Press.

67. Ohizumi H, Yoshioka M, Mori K, Miyazaki N. 1998 Stomach contents of common dolphins (Delphinus delphis ) in the pelagic western north Pacific. *Mar. Mammal Sci.* **14**, 835–844.

68. Westgate AJ, Read AJ. 2007 Reproduction in short-beaked common dolphins (Delphinus delphis) from the western North Atlantic. *Mar. Biol.* **150**, 1011–1024. (doi:10.1007/s00227-006-0394-1)

69. Danil K, Chilvers S, Ellis H, Perrin WF, Ripley BJ. 2004 Growth and reproduction of female short-beaked common dolphins, Delphinus delphis, in the eastern tropical Pacific. *Mar. Sci.* , 192. (doi:10.1139/z06-188)

70. Whitehead H. 2003 *Sperm Whales: Social Evolution in the Ocean*. London: The Univerisity of Chicago Press Ltd.

71. Best PB, Canham PAS, Macleod N. 1984 Patterns of reproduction in sperm whales, Physeter macrocephalus. *Rep. Int. Whal. Comm.* , 51–79.

72. Ohsumi S. 1965 Reproduction of the sperm whale in the north-west Pacific. *Sci. Reports Whales Res. Institute, Tokyo* **19**, 1–35.

73. Whitehead H, Antunes R, Gero S, Rendell L. 2012 Multilevel societies of female sperm whales (Physeter macrocephalus ) in the Atlantic and Pacific : why are they so different ? *Int. Hournal Primatol.* **33**, 1142–1164. (doi:10.1007/s10764-012-9598-z)

74. Gero S *et al.* 2014 Behavior and social structure of the sperm whales of Dominica , West Indies. *Mar. Mammal Sci.* **30**, 905–922. (doi:10.1111/mms.12086)

75. Gero S, Bøttcher A, Whitehead H, Madsen PT. 2016 Socially Segregated, Sympatric Sperm Whale Clans in the Atlantic Ocean. *R. Soc. Open Sci.* (doi:10.1098/rsos.160061)

76. Perrin WF. 2002 Spinner Dolphin *Stenella longirostris*. In *Encyclopedia of Marine Mammals* (eds WF Perrin, B Würsig, JGM Thewissen), pp. 1174–1178. London: Academic Press.

77. Dolar MLL, Walker WA, Kooyman GL, Perrin WF. 2003 Comparative feeding ecology of Spinner Dolphins (*Stenella longirostris*) and Fraser’s Dolphin (*Lagenodelphis hosei*) in the Sulu Sea. *Mar. Mammal Sci.* **19**, 1–19.

78. Perrin WF, Holts DB, Miller RB. 1977 Growth and reproduction of the eastern spinner dolphin, a geographical form of Stenella longirostris in the Eastern Tropical Pacific. *Fish. Bull.* **75**, 725–750.

79. Gerrodette T, Forcada J. 2005 Non-recovery of two spotted and spinner dolphin populations in the eastern tropical Pacific Ocean. *Mar. Ecol. Prog. Ser.* **291**, 1–21.

80. Andrews KR, Karczmarski L, Au WWL, Rickards SH, Vanderlip CA, Bowen BW, Gordon Grau E, Toonen RJ. 2010 Rolling stones and stable homes: Social structure, habitat diversity and population genetics of the Hawaiian spinner dolphin (Stenella longirostris). *Mol. Ecol.* **19**, 732–748. (doi:10.1111/j.1365-294X.2010.04521.x)

81. Archer II FI. 2002 Striped Dolphin *Stenella coeruleoba* . In *Encyclopedia of Marine Mammals* (eds WF Perrin, B Würsig, JGM Thewissen), pp. 1201–1203. London: Academic Press.

82. Archer II FI, Perrin WF. 1999 Stenella coeruleoalba. *Mamm. Species* **603**, 1–9.

83. Miyazaki N, Nishiwaki M. 1978 School structure of the striped dolphin off the Pacific coast of Japan. *Sci. Reports Whales Res. Institute, Tokyo* **30**, 65–115.

# Supplementary 6: Origin of data used in this study

Origins of age-specific corpora data used in this study.

| **Species** | **Family** | **Data** | **N. Whales** |
| --- | --- | --- | --- |
| **Atlantic white-sided dolphin**  *Lagenorhynchus acutus* | Delphinidae | Stranding events. NE USA. 1973-1974 | 33 |
| **Baird’s beaked whale**  *Berardius bairdii* | Ziphiidae | Japanese drive fishery 1976 + 1985-1988 | 43 |
| **Beluga whale**  *Delphinapterus leucas* | Monodontidae | Alaskan aboriginal subsistence hunts 1987-1998 | 124 |
| **Common bottlenose dolphin**  *Tursiops truncatus*  (3 populations) | Delphinidae | 1. Japanese drive fishery in the Korean Strait 1973-1983 2. Japanese drive fishery off Japan’s Pacific coast1973-1983 3. Accidentally killed in anti-shark nets South Africa 1980-1987 | 114  97  43 |
| **False killer whale**  *Pseudorca crassidens* | Delphinidae | Japanese drive fishery 1979-1980. (South African mass stranding data not included in the analysis) | 54 |
| **Harbour porpoise**  *Phocoena phocoena* | Phocoenidae | Whales stranded in the UK. 1990-2012 | 79 |
| **Long-finned pilot whale**  *Globicephala melas* | Delphinidae | Faroe Island drive fishery 1986-1988 | 613 |
| **Melon-headed whale**  *Peponocephala electra* | Delphinidae | 3 mass stranding’s in Japan in 1982, 2001 and 2002 | 55 |
| **Narwhal**  *Monodon monoceros* | Monodontidae | Greenland aboriginal subsistence hunts 2007-2010 | 53 |
| **Northern right-whale dolphin**  *Lissodelphis borealis* | Delphinidae | Bycatch in Japanese squid drift nets 1990-1991 | 87 |
| **Pantropical spotted dolphin**  *Stenella attenuata* | Delphinidae | Japanese drive fishery 1970-1975 | 116 |
| **Short-finned pilot whale**  *Globicephala macrorhynchus* | Delphinidae | Japanese drive fishery 1965-1972 | 188 |
| **Short-beaked common dolphin**  *Delphinus delphis* | Delphinidae | Stranding’s and incidental mortality from fishing. Atlantic France 1999-2005 | 123 |
| **Sperm Whale**  *Physeter macrocephalus* | Physeteridae | Japanese commercial whaling 1960-1962 | 857 |
| **Spinner Dolphin**  Stenella longirostris | Delphinidae | Incidental mortality from Eastern Pacific Yellowfin tuna fishery (1968-1975). | 168 |
| **Striped Dolphin**  *Stenella coeruleoalba* | Delphinidae | Japanese drive fishery 1970-1975 | 142 |
